# Introducing Chlorido Cerium Complexes with the Methoxy Kläui Ligand [Co(η^5^-C_5_H_5_){P(O)(OMe)_2_}_3_]^−^ †

**DOI:** 10.3390/molecules31162865

**Published:** 2026-08-17

**Authors:** Peter Ferber, Christoph Janiak

**Affiliations:** Institut für Anorganische Chemie und Strukturchemie, Heinrich-Heine-Universität Düsseldorf, D-40204 Düsseldorf, Germany; peter.ferber@uni-duesseldorf.de

**Keywords:** cerium, crystal structure, tripod ligands, Kläui ligands, shape calculation, bond valence sum, metal–organic chemistry

## Abstract

The treatment of Ce(OH)_4_ in thionyl chloride and dimethoxyethane (DME) resulted in the formation of a highly unstable red Ce(IV) complex with the composition [CeCl_4_(DME)_2_] (**1**). From the reaction of **1** with the Na[Co(η^5^-C_5_H_5_){P(O)(OMe)_2_}_3_] (NaL_OMe_) salt containing the anionic tripodal Kläui ligand η^5^-cyclopentadienyltris(dimethylphosphonato)cobaltate(III), the two new cerium(III) complexes [Ce(L_OMe_)_2_(H_2_O)_2_]Cl·2.5H_2_O (**2**), 1D-[Ce(μ-Cl)(L_OMe_)(H_2_O)_3_]Cl (**3**) and the cerium(IV) complex [CeCl_2_(L_OMe_)_2_]·acetone (**4**) were obtained and structurally characterized. The complexes **3** and **4** are the first cerium complexes featuring the L_OMe_ ligand together with chlorido ligands. Due to the potential exchange of the chloride counterions with other ligands in **2**−**4**, these complexes could serve as precursors in molecular cerium coordination chemistry. The assigned cerium oxidation states are supported by the bond valence sum (BVS) method. The ^31^P-NMR spectra with δ(^31^P) = 160.20 ppm (**2**) and 159.43 ppm (**3**) are diagnostic of cerium(III).

## 1. Introduction

Cerium with its +III and +IV oxidation states has a wide range of established and emerging applications, spanning the water-gas shift reaction [1], catalytic oxidation processes [2], organic synthesis [3], and more recent developments in solar energy to fuel conversion [4]. In addition, cerium-based materials attract increasing attention in biomedical contexts, where they can contribute to advanced chemo- and immunotherapeutic strategies [5]. In particular, a growing number of studies have shown how Ce complexes can be used in many areas of materials science. Their special electronic structure and coordination chemistry have made them useful in a range of applications. These include organic light-emitting diodes [6] and radical for durability enhancement in proton-exchange membrane fuel cells and organic photovoltaics [7]. They are also used as atomic layer deposition precursors [8] and for fluorescence-based chemical sensing [9].

**Scheme 1:**
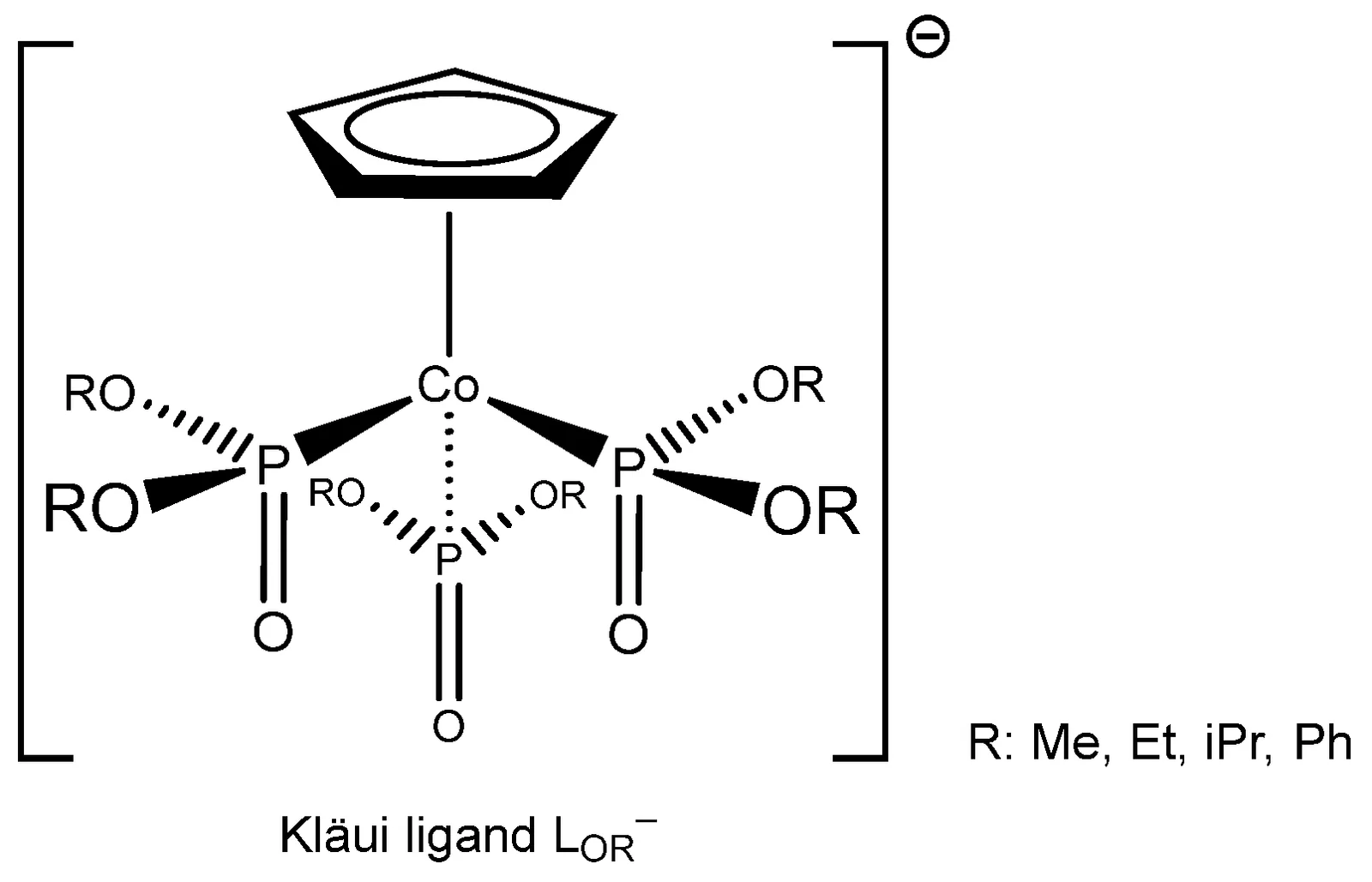
Schematic presentation for the monoanionic tripodal Kläui ligands [Co(η^5^-C_5_H_5_){P(O)(OR)_2_}_3_]^−^ (abbreviated as L_OR_ with R = Me, Et, iPr, Ph).

Monoanionic tripodal ligands of the type [Co(η^5^-C_5_H_5_){P(O)(OR)_2_}_3_]^−^ (abbreviated as L_OR_ with R = Me, Et, iPr, Ph) are commonly known as Kläui ligands (Scheme 1). These six-electron, oxygen-donor ligands can to some extent be regarded as analogs of the cyclopentadienyl ligand C_5_H_5_^−^ (Cp^−^) [10]. On the other hand, the ligand is a relatively weak-field and π-donating donor with bonding characteristics comparable to water or dimethyl sulfoxide yet exhibit enhanced stability [10]. Owing to these properties, they effectively stabilize metals in high oxidation states across the periodic table and have been widely employed as models for metal–oxygen environments in catalysis [11]. At the same time, their good solubility and hydrolytic stability make them useful mimics of facially coordinated aqua ligands in organometallic systems [12,13,14].

Due to the intriguing properties of cerium and the stabilizing effect of Kläui ligands, their combination is of interest. The formation of a well-defined coordination environment at the cerium center by the tripodal Kläui ligand, and its stabilizing effect, makes it possible to investigate and compare the reactivity of other ligands at cerium in the presence of the inactive Kläui ligand. For instance, it has been reported that Ce-Kläui-carboxylate and Ce-Kläui-carbonate-complexes undergo hydrogen-atom transfer reactions as well as visible-light-induced decarboxylation. Furthermore, the aforementioned complexes have been employed as catalyst precursors for the photocatalytic decarboxylative oxygenation of arylacetic acids under aerobic conditions [15]. The presence of a Kläui ligand in the coordination shell and the variation in the side groups on the phosphonates enables the tuning of the hydrophobicity and steric demand around the cerium center.

The Kläui ligand-cerium complexes with R = Et have already demonstrated potential in various other fields of application, particularly in catalysis. Such systems have been employed in the aerobic oxidation of cumene to 2-phenyl-2-propanol and acetophenone [16], and in the oxidation of substituted thioanisoles [17]. Both Ce(IV) and Ce(III) complexes have been shown to be active in these transformations.

In particular, the Kläui ligand-cerium(IV)-chloride complexes [CeCl_3_(L_OEt_)] [18] and [CeCl_2_(L_OEt_)_2_] [19] (L_OEt_ = η^5^-cyclopentadienyltris(diethylphosphonato)cobaltate(III), [Co(η^5^-C_5_H_5_){P(O)(OEt)_2_}_3_]^−^) are of significant importance, as they serve as suitable precursors for the synthesis of Ce-oxo clusters [18] and enable the incorporation of addition metals such as Mo [17], Re [17], V [17,20], Mn [16], Ru [20] and Os [20]. Furthermore, the chloride ligands can be substituted by anions such as IO_4_^−^, S_2_O_8_^−^, and IO_3_^−^ [21]. Replacement of chloride by alkoxide ligands can promote oxidative C–C coupling reactions, especially when the alkoxide corresponds to 2,4-di-tert-butylphenol [22]. Moreover, when the aryloxide ligand is 2,4,6-tri-tert-butylphenol, oxidative transformations of these ligands can also occur [23].

Although the Kläui ligand-cerium-chloride complexes represent highly versatile precursors, only the two complexes [CeCl_3_(L_OEt_)] [18] and [CeCl_2_(L_OEt_)_2_] [19], containing exclusively chloride and Kläui ligands, have been reported in the Cambridge Structure Databank (CSD) [24]. Due to the diverse potential applications of cerium coordination complexes, there is significant interest in the synthesis of analogous cerium-chloride complexes with other Kläui ligands having for example the methoxy substituent on phosphorus, [Co(η^5^-C_5_H_5_){P(O)(OMe)_2_}_3_]^−^ (L_OMe_). Consequently, this work addressed the synthesis of novel, Kläui ligand-cerium-chloride complexes with L_OMe_.

## 2. Results and Discussion

**Scheme 2:**
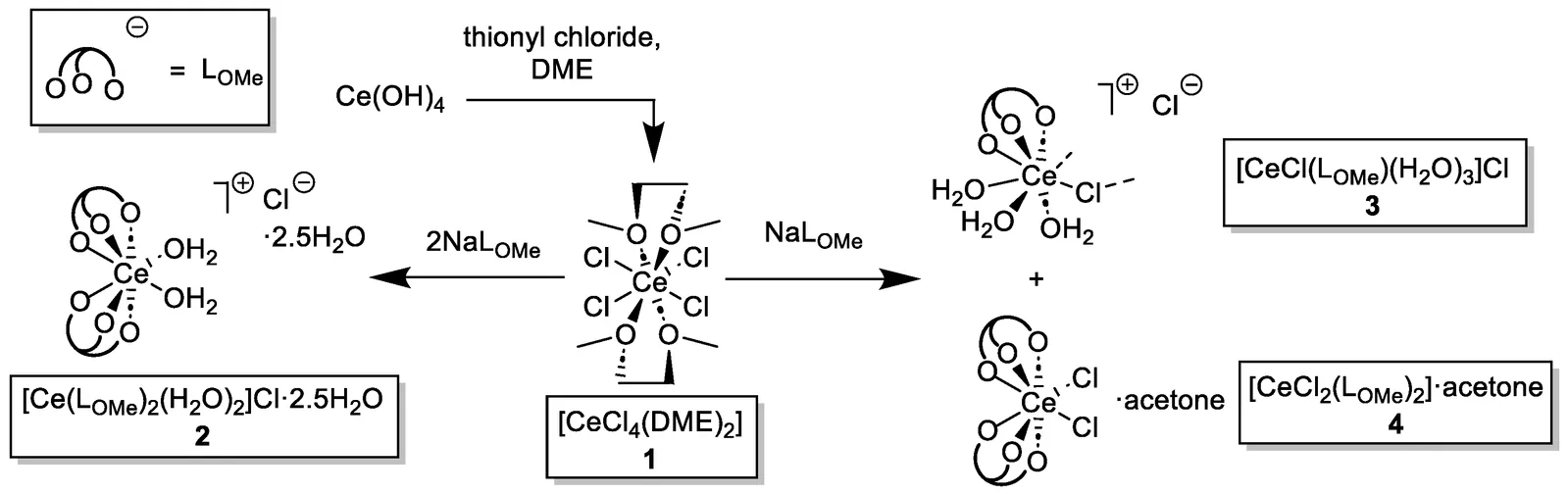
Formation of the complex [CeCl_4_(DME)_2_] (**1**) as starting material for the Kläui ligand complexes **2**–**4** (L_OMe_ = [Co(η^5^-C_5_H_5_){P(O)(OMe)_2_}_3_]^−^, DME = dimethoxyethane).

The treatment of Ce(OH)_4_ with thionyl chloride in dimethoxyethane (DME) yielded a dark red solid of formula [CeCl_4_(DME)_2_] (**1**) (Scheme 2, Figure S1), which was isolated and handled under inert conditions. The intense red color indicates a Ce(IV) complex. However, this complex is not stable under air due to its strongly hygroscopic nature.

### 2.1. Synthesis of [CeCl_4_(DME)_2_] (***1***)

It is observed that, within minutes upon exposure to the atmosphere, the red color of [CeCl_4_(DME)_2_] disappears and a white solid forms within the resulting pasty material which was identified as cerium(III) chloride heptahydrate (CeCl_3_·7H_2_O) by powder X-ray diffraction (Figure S2).

Even crystals of [CeCl_4_(DME)_2_] coated with perfluorinated oil and maintained under a nitrogen stream at 150 K degraded within two hours, which was clearly evident during the single-crystal X-ray diffraction (SCXRD) measurement. Nevertheless, the structure of **1** could be successfully determined. Based on these data, the bond valence sum (BVS) analysis (see below) supports the assignment as tetravalent cerium.

**Figure 1:**
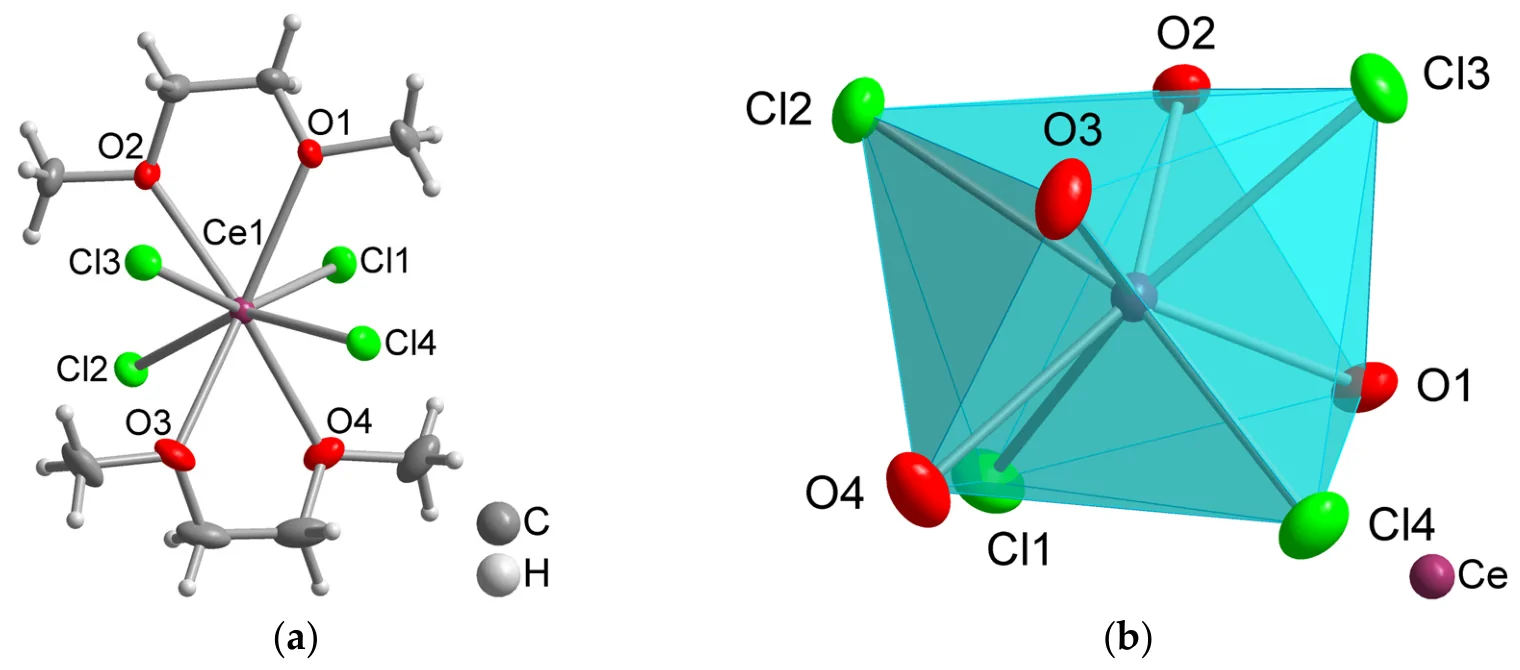
(**a**) Asymmetric unit in the structure of [CeCl_4_(DME)_2_] (**1**) (50% thermal ellipsoids, H atoms with arbitrary radii); (**b**) coordination polyhedron of cerium. Bond lengths around Ce are given in Table 1, bond angles in Table S6 (see Figure S9 for the packing diagram).

In the structure of **1,** the asymmetric unit comprises one central cerium atom, two dimethoxyethane (DME) molecules and four chloride ligands (Figure 1). This gives a coordination number of eight for the Ce atom.

The Ce–Cl bond lengths range from 2.5795 to 2.6107 Å, while the Ce–O distances lie between 2.5047 and 2.603 Å (Table 1). Accordingly, the Ce–Cl distances are slightly shorter than those reported for the also eight-coordinate cerium(III) compound [Ce_2_Cl_6_(DME)_4_] [25] (Ce–Cl: 2.7280–2.9269 Å; Ce–O: 2.5084–2.666 Å) as well as for the cerium(IV) compound (N(CH_3_)_4_)_2_[CeCl_4_(NO_3_)_2_] [26] (Ce–Cl: 2.6496 Å; Ce–O: 2.5013 Å), whereas the Ce–O distances in **1** are longer compared to those in (N(CH_3_)_4_)_2_[CeCl_4_(NO_3_)_2_].

Furthermore, the cis-Cl–Ce–Cl bond angles in **1** range from 84.44° to 105.79°, while the O–Ce–O angles within the respective DME ligands are 63.82° and 65.44° (Table S6). The cis-Cl–Ce–Cl angles differ significantly from those observed in the dinuclear [Ce_2_Cl_6_(DME)_4_] structure (72.46° and 85.82°) [25] In contrast, the O–Ce–O angles of the DME ligands remain largely unchanged due to the structural constraints of the DME backbone, with values of approximately 63°. The cis-Cl–Ce–Cl angles in the Ce(IV) compound (N(CH_3_)_4_)_2_[CeCl_4_(NO_3_)_2_] are exactly 90° by symmetry.

A continuous shape measure (CShM) analysis of the coordination sphere of the Ce atoms was performed with the SHAPE V2.1 software [27]. In this approach, an ideal polyhedron has a shape measure of S = 0, while increasing S values indicate increasing distortion from the ideal geometry. Complex **1** shows a preference for a square antiprismatic shape (SAPR), based on: S(SAPR) = 1.37, S(triangular dodecahedron (TDD)) = 1.98, and S(biaugmented trigonal prism (BTPR)) = 1.71. If the S values are not close to zero, the structure can lie between two ideal geometries. This can be determined by the interconversion analysis with a generalized coordinate and the deviation from the path between these two ideal geometries. The interconversion analysis reveals a significant generalized coordinate for TDD → SAPR = 83% and for SAPR → TDD = 69%, and large deviations from the ideal paths (SAPR, TDD) = 52% and (SAPR, BTPR) = 64%. Since there are large deviations from the indicated paths, the structure is not exactly on the direct path between two ideal shapes. The coordination geometry is best described as a distorted square antiprism, due to the lowest shape Measure parameter (S) and the higher interconversion coordinate in direction to the SAPR-shape, with contributions from triangular dodecahedral (TDD) and biaugmented trigonal prismatic (BTPR) geometries. In comparison, the polyhedron of [Ce_2_Cl_6_(DME)_4_] (Figure S17) can be described as a distorted triangular dodecahedron (S(TDD) = 0.91) and the geometry of the Ce in (N(CH_3_)_4_)_2_[CeCl_4_(NO_3_)_2_] (Figure S18) is best described as a gyrobifastigium, which is distorted by the two nitrate anions, even if the CShM is high (S(Johnsons gyrobifastigium J26) = 4.24).

### 2.2. Synthesis of [Ce(L_OMe_)_2_(H_2_O)_2_]Cl·2.5H_2_O (***2***)

**Figure 2:**
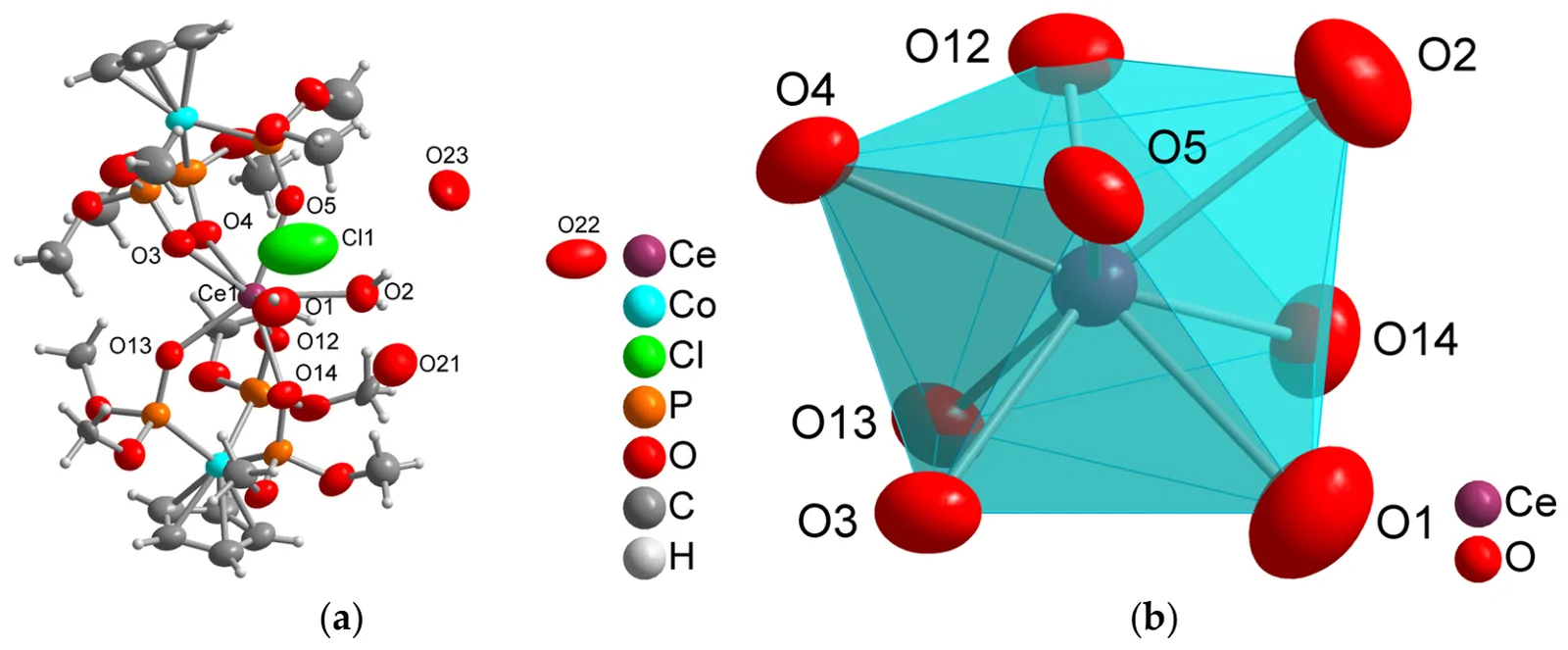
(**a**) Asymmetric unit in the structure of [Ce(L_OMe_)_2_(H_2_O)_2_]Cl·2.5H_2_O (**2**) (50% thermal ellipsoids, H atoms with arbitrary radii); (**b**) coordination polyhedron of cerium. A disorder of three of the phosphonate groups is not shown here for clarity (see Figure S11). Bond lengths around Ce are given in Table 2, bond angles in Table S10.

The reaction of **1** with two equivalents of NaL_OMe_ in acetone resulted in the formation of intense green crystals (Scheme 2, Figure S1) upon slow solvent evaporation over several days. The crystals were analyzed as [Ce(L_OMe_)_2_(H_2_O)_2_]Cl·2.5H_2_O with Ce(III) by single-crystal X-ray diffraction. The ^31^P-NMR also indicates the presence of a trivalent cerium with a single peak at 160.20 ppm (Figure S7), since the ^31^P resonances at 115-125 ppm are diagnostic for tetravalent cerium in Ce-L_OEt_ complexes and resonances at 150-160 ppm indicate trivalent cerium in Ce-L_OEt_ complexes [23]. The relatively large linewidths in the NMR spectra of **2** are typical of paramagnetic Ce(III) complexes and are due to an increased relaxation caused by the unpaired electron on the Ce(III) center. The presence of a trivalent cerium is also underlined by the BVS method calculated by the results of the SCXRD measurement (see below). The asymmetric unit in the structure of [Ce(L_OMe_)_2_(H_2_O)_2_]Cl·2.5H_2_O corresponds to the formula unit and contains an eight-coordinated central cerium atom, which binds two Kläui ligands and two aqua ligands (Figure 2). In addition, there is a disordered chloride counter anion and two and a half crystal water molecules. The aqua ligands build hydrogen bonds to the disordered chloride anion (Figures S12 and S13).

**Table 2:** Bond lengths around cerium in **2** ^a^.

	Bond Length [Å]
Ce1—O1	2.546 (11)
Ce1—O2	2.583 (9)
Ce1—O3	2.479 (7)
Ce1—O4	2.447 (8)
Ce1—O5	2.419 (8)
Ce1—O12	2.395 (8)
Ce1—O13	2.414 (7)
Ce1—O14	2.484 (7)

^a^ Bond angles around Ce are given in Table S10.

The structure of **2** can be compared best with the structures of the two Ce(IV) complexes [Ce(L_OEt_)_2_(H_2_O)_2_](OTf)_2_·H_2_O (OTf = CF_3_SO_3_^−^) [28] and [Ce(L_OEt_)_2_(H_2_O)_2_](BF_4_)_2_ [23] as well as to the Ce(III) complex in [Ce(L_OEt_)_2_(H_2_O)_2_](L_OEt_) [28], since all of these compounds feature two Kläui and two aqua ligands, giving rise to eight-coordinate cerium centers surrounded exclusively by oxygen atoms.

The Ce–O distances of the aqua ligands in **2** range from 2.546 to 2.583 Å, whereas the Ce–O distances to the Kläui ligands lie between 2.395 and 2.484 Å. The distances in the above Ce(IV) from the literature are shorter, reflecting the higher charge and smaller size of the Ce(IV) centers. In Ce(L_OEt_)_2_(H_2_O)_2_](OTf)_2_·H_2_O, the Ce–O(aqua) distances are 2.475 and 2.466 Å; the Ce–O(L_OEt_) distances span 2.226–2.310 Å [28]. In [Ce(L_OEt_)_2_(H_2_O)_2_](BF_4_)_2_, the Ce–O(aqua) distances are between 2.505 and 2.506 Å, Ce–O(L_OEt_) 2.265–2.293 Å [23].

Comparison with the Ce(III) complex [Ce(L_OEt_)_2_(H_2_O)_2_](L_OEt_) shows only minor variations, with Ce–O(aqua) distances of 2.531 and 2.579 Å and Ce–O(L_OEt_) distances of 2.404–2.516 Å. The most noticeable difference concerns the O–Ce–O angles between the aqua ligands: in **2**, this angle is 80.8°, compared to 73.8° in Ce(L_OEt_)_2_(H_2_O)_2_](OTf)_2_·H_2_O [28] 77.0° in [Ce(L_OEt_)_2_(H_2_O)_2_](BF_4_)_2_ [23], and the Ce(III) complex [Ce(L_OEt_)_2_(H_2_O)_2_][L_OEt_] with 71.3° [28].

The coordination polyhedron of Ce in **2** fits best as a square antiprism, since S(SAPR) = 0.65 is clearly lower than S(TDD) = 1.93 and S(BTPR) = 1.45. This is supported by the generalized coordinates for the interconversions, TDD → SAPR = 82% and SAPR → TDD = 48%, plus a moderate deviation from the path (SAPR, TDD) = 30% and also by the interconversion from BTPR → SAPR with 80% and SAPR → BTPR with 54% and the deviation from the path with 33%. Thus, the SAPR polyhedron in **2** is slightly distorted towards the BTPR and TDD geometry.

In comparison, the Ce(IV) literature complexes feature an even more ideal SAPR, with S(SAPR) = 0.24 for [Ce(L_OEt_)_2_(H_2_O)_2_](OTf)_2_·H_2_O (Figure S19) and S(SAPR) = 0.24 for [Ce(L_OEt_)_2_(H_2_O)_2_](BF_4_)_2_ (Figure S20). [Ce(L_OEt_)_2_(H_2_O)_2_][L_OEt_] (Figure S21) shows a better fit with a biaugmented trigonal prism geometry for Ce(III) (S(BTPR) = 1.46).

The experimental PXRD pattern of **2** agrees well with the simulated pattern with respect to peak positions, confirming phase purity (Figure S3). Differences in relative intensities, namely the attenuation of the (110) (5.97° 2θ) and (020) (8.47° 2θ) reflections and the increased intensity of the (101) (9.52° 2θ) reflection, are attributed to preferred orientation on the flat sample holder with Bragg–Brentano geometry. Simulations employing a March–Dollase correction along [101] reproduce these features, supporting this assignment (Figure S3). No additional reflections indicative of crystalline impurities are observed.

### 2.3. Synthesis of 1D-[Ce(μ-Cl)(L_OMe_)(H_2_O)_3_]Cl (***3***) and [CeCl_2_(L_OMe_)_2_]·acetone (***4***)

Treatment of **1** with only one equivalent NaL_OMe_ in acetone yielded slightly yellowish crystals (Figure S1), which analyzed as 1D-[Ce(μ-Cl)(L_OMe_)(H_2_O)_3_]Cl (**3**) as the main product, alongside trace amounts of intensely red crystals of [CeCl_2_(L_OMe_)_2_]·acetone (**4**). The intense red color of **4** (Figure S1) indicates the presence of a tetravalent cerium atom, but due to the low yield it could be only investigated by single-crystal X-ray diffraction (see below). At the same time, compound **3** could be obtained in relatively pure form by washing with ice-cold water which removed the intense red color of the side product **4**. This was also proven by the very good match between the measured PXRD pattern (Figure S4) and the simulated pattern from the SCXRD measurement. The pale yellowish color of **3** indicates a trivalent cerium atom. The ^31^P-NMR of **3** with a chemical shift at 159.43 ppm (Figure S8) also supports the trivalent state of the cerium. As is typical for paramagnetic Ce(III) complexes, the NMR signals of **3** display relatively broad linewidths due to the enhanced relaxation caused by the unpaired electron at the Ce(III) center. The oxidation states of the cerium atoms in **3** and **4** could also be derived by the BVS analysis (see below).

**Figure 3:**
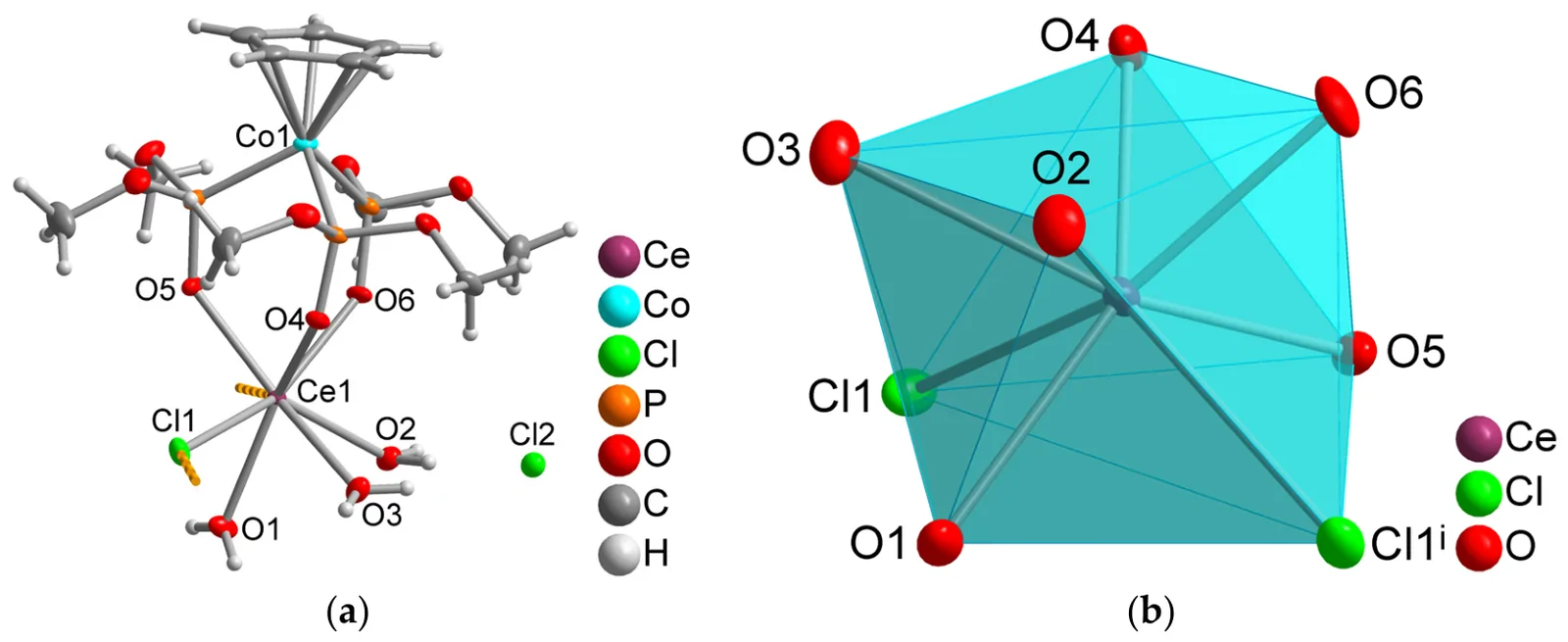
(**a**) Asymmetric unit in the structure of 1D-[Ce(µ-Cl)(L_OMe_)(H_2_O)_3_]Cl (**3**) (50% thermal ellipsoids, H atoms with arbitrary radii), (**b**) coordination polyhedron of cerium. Symmetry code: (i) y + 1/2, x − 1/2, z − 1/2. Bond lengths around Ce are given in Table 3, bond angles in Table S13.

**Figure 4:**
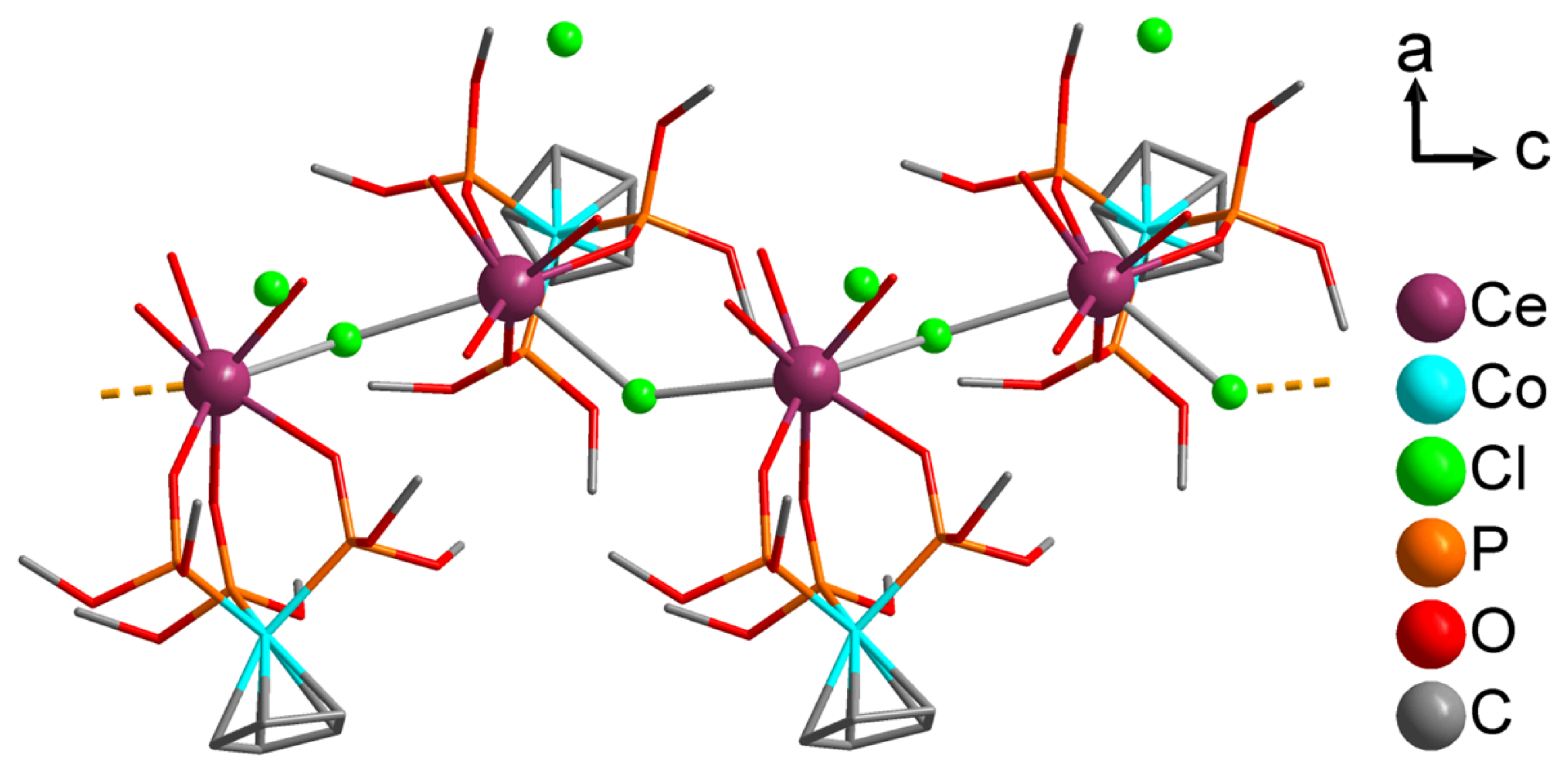
1D-chain structure in [CeCl(L_OMe_)(H_2_O)_3_]·Cl (**3**), with the Kläui ligand as a wireframe model (H atoms are omitted for clarity).

**Figure 5:**
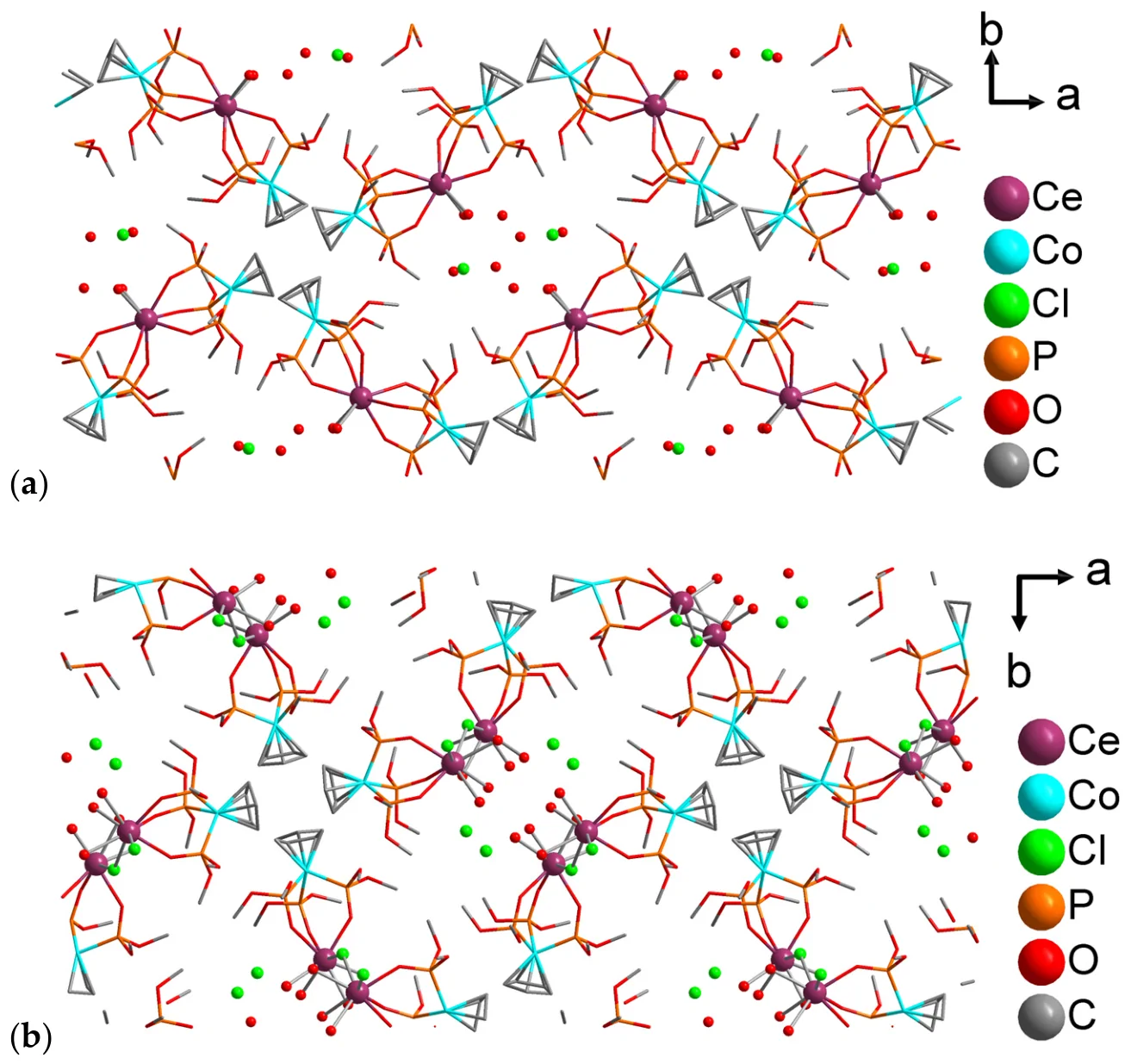
Packing diagrams with two unit cells of (**a**) [Ce(L_OMe_)_2_(H_2_O)_2_]Cl·2.5H_2_O (**2**); (**b**) [CeCl(L_OMe_)(H_2_O)_3_]·Cl (**3**), both viewed along the c-axis to show the similar orientation of hydrophobic and hydrophilic regions (Kläui ligand as a wireframe model, H atoms are omitted for clarity; see Figures S10 and S14 for ball-and-stick representations).

The asymmetric unit in the structure of 1D-[Ce(μ-Cl)(L_OMe_)(H_2_O)_3_]Cl (**3**) corresponds to the formula unit which includes a cerium atom, coordinated with one Kläui ligand L_OMe_, a bridging chlorido ligand and three aqua ligands (Figure 3). An additional, non-coordinated chloride counter-anion (Cl2) is hydrogen-bonded to the water molecules (Figure S15). The cerium atoms are connected through the coordinated Cl1 atom, which results in the formation of a 4_2_-helical chain structure along the c-axis (Figure 4 and Figure 5b).

**Table 3:** Bond lengths around cerium in **3** ^a^.

	Bond Length [Å]
Ce1—Cl1	2.9137 (16)
Ce1—Cl1^i^	2.9552 (18)
Ce1—O1	2.513 (5)
Ce1—O2	2.592 (5)
Ce1—O3	2.510 (5)
Ce1—O4	2.479 (5)
Ce1—O5	2.370 (4)
Ce1—O6	2.369 (4)

^a^ Bond angles around Ce are given in Table S13. Symmetry code: (i) y + 1/2, x − 1/2, z − 1/2.

The Ce(III) structure of **3** can be compared with the structures of two Ce(IV) complexes [CeCl(L_OEt_)_2_(OC_6_H_3_Cl_2_-2,4)] [23] and [CeCl(L_OEt_)_2_(OC_6_H_3_Cl_3_-2,4,6)] [23], as these feature eight-coordinate cerium centers with both chloride and oxygen donors in the coordination sphere, albeit with a different, namely Ce(IV) oxidation state. To the best of our knowledge, no Ce(III) complex with a non-coordinating chloride and a Kläui ligand is reported in the literature. The complex [Ce_2_Cl_6_(DME)_4_] can also be compared with **3**, because as it also exhibits Ce(III) centers bridged by μ-chlorido ligands.

In **3**, the Ce–Cl bond lengths are 2.9137 and 2.9552 Å, the Ce–O(L_OMe_) distances range from 2.370 to 2.479 Å, the Ce–O(H_2_O) distances lie between 2.510 and 2.592 Å and are therefore only marginally longer than those observed in **2**. In contrast, the Ce–Cl bond lengths in the cerium(III) complex **3** are expectedly longer than those reported for [CeCl(L_OEt_)_2_(OC_6_H_3_Cl_3_-2,4,6)] (2.737 Å) and [CeCl(L_OEt_)_2_(OC_6_H_3_Cl_2_-2,4)] (2.7544 Å) with their Ce(IV) oxidation state [23]. Likewise, the Ce–O bond lengths in [CeCl(L_OEt_)_2_(OC_6_H_3_Cl_2_-2,4)] and [CeCl(L_OEt_)_2_(OC_6_H_3_Cl_3_-2,4,6)], which range from 2.327 to 2.349 Å and from 2.320 to 2.370 Å, respectively, are slightly shorter than in **3**.

Notably, the Ce–Cl–Ce bond angle in **3** is 135.82°, which is significantly larger than the corresponding bridging-chloride angle of 107.54° observed in [Ce_2_Cl_6_(DME)_4_] [25].

For **3**, the lowest CShM value is for SAPR (S(SAPR) = 1.10), while S(TDD) = 2.87 and S(BTPR) = 1.87 show more distortion. The generalized coordinate for the interconversion TDD→SAPR yields 100% and SAPR→TDD 62%, but with a significant deviation from the path (SAPR, TDD) of 62%, which indicates that the structure is quite far from the ideal SAPR→TDD path. The path between SAPR and BTPR also gives a deviation with 60%, but also a high interconversion coordinate BTPR→SAPR with 91% and a significant lower coordinate for SAPR→BTPR of 70%. Together, this suggests **3** is a distorted eight-coordinate structure with SAPR character and distortion to BTPR and TDD geometry. In comparison the polyhedron of cerium in [CeCl(L_OEt_)_2_(OC_6_H_3_Cl_2_-2,4)] could be described as triangular dodecahedron (Figure S22) (S(TDD) = 1.11) and [CeCl(L_OEt_)_2_(OC_6_H_3_Cl_3_-2,4,6)] as a less distorted square antiprism (Figure S23) (S(SAPR) = 0.95).

It is interesting to note that the packing in the structure of **3** with space group P4_2_bc is similar to the structure of **2** in the lower symmetry space group P2_1_2_1_2 along the crystallographic c-axis (Figure 5). The hydrophobic C_5_H_5_ ligands are oriented towards each other, while the hydrophilic aqua ligands and the methoxy groups surround the channels with the chloride counter-anions which in the structure of **2** also contain water solvent molecules.

**Figure 6:**
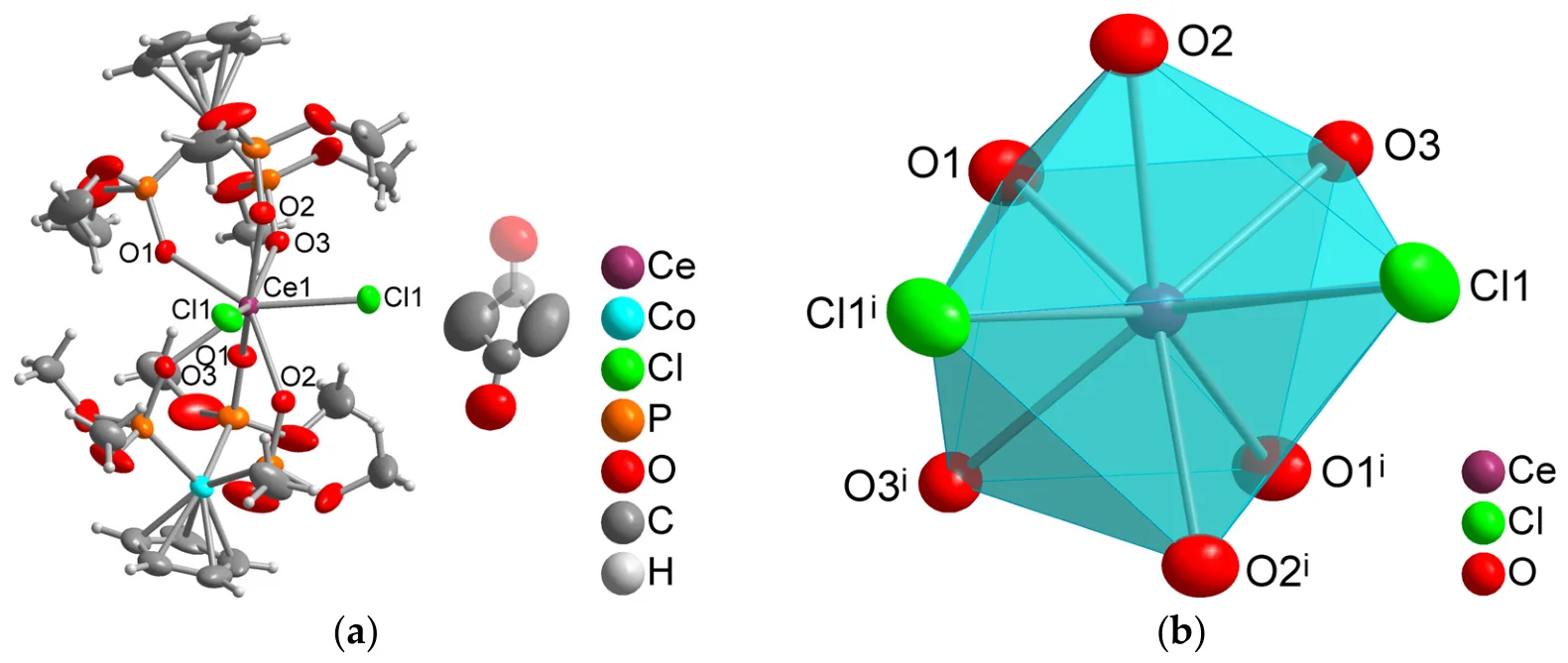
(**a**) Extended asymmetric unit in the structure of [CeCl_2_(L_OMe_)_2_]·acetone (**4**) (50% thermal ellipsoids, H atoms with arbitrary radii and a disordered acetone molecule); (**b**) coordination polyhedron of cerium. Symmetry code: (i) −x + 1, −y + 1, z. Bond lengths around Ce are given in Table 4, bond angles in Table S15.

**Figure 7:**
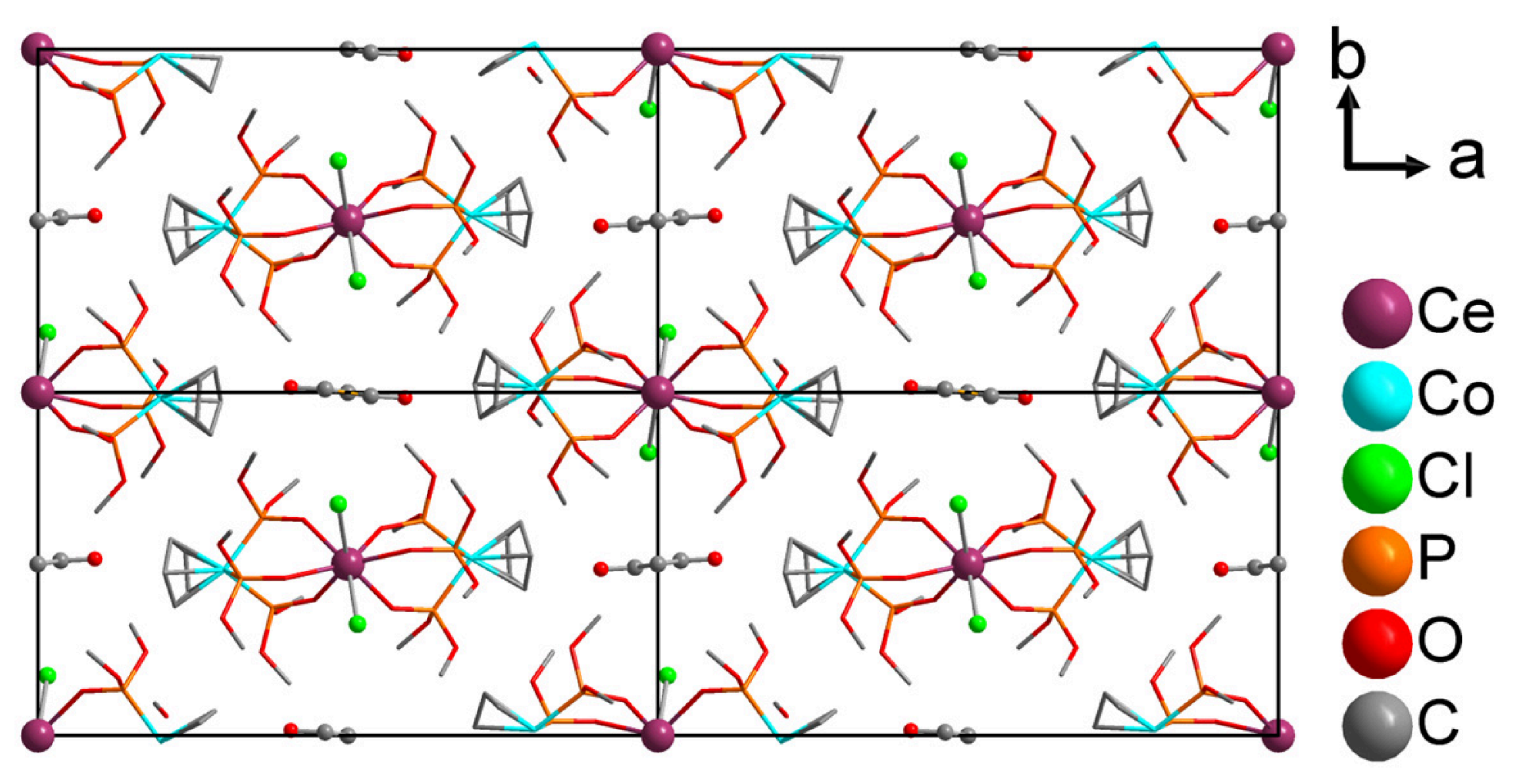
Packing diagram with four unit cells of [CeCl_2_(L_OMe_)_2_]·acetone (**4**) with view along the c-axis (Ce, Co, O, Cl in ball and stick model and ligand in capped stick model, and H atoms are omitted for clarity; see Figure S16 for ball-and-stick representation).

The trace-amount side product **4**, which forms together with **3** (Scheme 1), was analyzed as [CeCl_2_(L_OMe_)_2_]·acetone with two coordinated Kläui ligands and two coordinated chlorido ligands at the eight-coordinate Ce(IV) center (Figure 6). In addition, there is a disordered acetone molecule with two positions around a C_2_-axis which fills the space between the complex molecules in the crystal packing (Figure 7). There is only half of the molecule in the asymmetric unit with a C_2_-axis bisecting the L-Ce-L and Cl-Ce-Cl angles.

**Table 4:** Bond lengths around cerium in **4** ^a^.

	Bond Length [Å]
Ce1—Cl1^i^	2.7128 (18)
Ce1—Cl1	2.7128 (18)
Ce1—O1^i^	2.329 (5)
Ce1—O1	2.329 (5)
Ce1—O2	2.336 (5)
Ce1—O2^i^	2.336 (5)
Ce1—O3	2.350 (5)
Ce1—O3^i^	2.350 (5)

^a^ Bond angles around Ce are given in Table S15. Symmetry code: (i) −x + 1, −y + 1, z.

Complex **4** is best compared to the coordination environment of [CeCl_2_(L_OEt_)_2_] [19], as the two Ce(IV) compounds differ only in the side groups in the Kläui ligand (methoxy vs. ethoxy). The Ce–Cl bond lengths in **4** (2.7128 Å) are a bit longer than those reported for the L_OEt_ literature compound (2.7090 and 2.7118 Å). The Ce–O bond distances in **4** from 2.329 to 2.350 Å, show less variation compared to the L_OEt_ reference compound (2.3171 to 2.3780 Å). The Cl–Ce–Cl bond angle in **4** is 97.33°, which is close to the 99.55° angle reported in [CeCl_2_(L_OEt_)_2_] [19].

The coordination environment around Ce in **4** is best described as a trigonal dodecahedron, with the lowest value S(TDD) = 0.67, while S(SAPR) = 2.77 and S(BTPR) = 2.92 are less suitable. The generalized coordinate for the interconversion SAPR → TDD is 99% and for TDD → SAPR it is 48%, along with 47% deviation to the path (SAPR, TDD), which confirms that the TDD shape dominates, though there is a noticeable distortion. The test on the interconversion BTPR→TDD shows a generalized coordinate of 104% while TDD → BTPR gives the generalized coordinate of 49% with a deviation of the path of 53%. This shows the structure deviates from the ideal path between shapes of TDD, BTPR and SAPR arrangement with a strong dominance of the triangular dodecahedral geometry. [CeCl_2_(L_OEt_)_2_] also shows TDD character with S(TDD) = 0.62 (Figure S24). More remarkable is that [CeCl_2_(L_OEt_)_2_] crystallizes in the monoclinic centrosymmetric space group P2_1_/c, in contrast to **4**, which crystallizes in the orthorhombic acentric space group P2_1_2_1_2.

The occurrence of the acentric space groups P4_2_bc (for **3**) and P2_1_2_1_2 (for **2** and **4**) is consistent with Kitaigorodski’s [29,30] description of molecular crystals where packings of irregularly shaped entities favor acentric over centrosymmetric arrangements for efficient space filling.

A bond valence sum (BVS) analysis based on Pauling’s model using the Ce–O/Cl bond lengths [31], supports the assigned oxidation states (Table 5) of the cerium atoms in compounds **1**–**4**, from the single-crystal X-ray refinements. Irrespective of whether the bond length parameters for Ce(III) and Ce(IV) were used, the BVS analysis gives oxidation states of IV for **1** and **4**, and oxidation states of III for **2** and **3** (Table 5). The BVS analysis was carried out with Equation (1) using R_0_(Ce(III)–O) = 2.112 Å [32], R_0_(Ce(III)–Cl) = 2.504 Å [33], R_0_ (Ce(IV)–O) = 2.076 Å [33], R_0_(Ce(IV)–Cl) = 2.46 Å [18], R_ij_ = bond length between atoms i and j, B = 0.37 Å [33].

Oxidation State = Σ exp((R_0_−R_ij_)/B) (1)

Since the dataset of eight coordinated tetravalent cerium complexes with chloride ligands is quite low, a recalculation of R_0_ using the distances of the coordination sphere of **1** would result in an R_0_(Ce(IV)–Cl) value of 2.489 Å to yield a valence sum of 4.000, with a B value of 0.37.

## 3. Materials and Methods

All chemicals were purchased from commercial suppliers and used as received unless otherwise stated (see SI for further information Table S1). The Kläui ligands were synthesized according to the literature [34].

NMR spectra were recorded on a Bruker Avance NEO evo—600 spectrometer (Bruker, Karlsruhe/Ettlingen, Germany) operating at 600 and 243 MHz for ^1^H and ^31^P, respectively. Chemical shifts (δ, ppm) were referenced to the residual proton solvent signal of CHCl_3_ (δ = 7.26 ppm) for ^1^H and to neat H_3_PO_4_ for ^31^P. Elemental analyses were conducted on a Perkin Elmer 2400 series II elemental analyzer (PerkinElmer, Waltham, MA, USA). Powder X-ray diffractograms (PXRD) were collected at room temperature on a Rigaku Mini-Flex 600 diffractometer (600 W, 40 kV, 15 mA) (Rigaku, Tokyo, Japan) using Cu K_α_ radiation (λ = 1.54182 Å). The experiments were performed with 0.01° steps in the range of 5–50° 2θ. Simulated PXRD patterns were obtained by MERCURY 2020.3.0. FT-IR spectra were recorded on a Bruker TENSOR 37 IR spectrometer (Bruker AXS, Karlsruhe, Germany) in KBr from 4000 to 400 cm^−1^.

### 3.1. Single-Crystal X-Ray Crystallography

Suitable single crystals were selected under a polarized-light microscope, covered in protective oil and mounted on a cryo-loop. A Rigaku XtaLAB Synergy S (Rigaku, Tokyo, Japan) four circle diffractometer with a hybrid pixel array detector and a PhotonJet X-ray source for CuK_α_ radiation (λ = 1.54184 Å) and MoK_α_ radiation (λ = 0.71073 Å) with a multilayer mirror monochromator was used to collect the single-crystal diffraction data. Data were obtained at 150.0 ± 0.1 K using ω-scans. Data reduction and absorption correction were performed via CrysAlisPro 1.171.41.90a [35]. The structures were solved by direct methods with SHELXT-2015), Full-matrix least-squares refinements on F^2^ were carried out using the SHELXL-2017/1 program package in Olex 2.1.5 [36,37,38]. The hydrogen atoms on C were positioned geometrically (with C–H = 0.95 Å for aromatic and aliphatic CH, C–H = 0.99 Å for CH_2_ and C–H = 0.98 Å for CH_3_) and refined using riding models (AFIX 43, AFIX 23 and AFIX 137) with U_iso_(H) = 1.2 U_eq_ (CH and CH_2_) and 1.5 U_eq_ (CH_3_). In **2** and **3** the hydrogen atoms (O1 and O2 in **2** and O1, O2 and O3 in **3**) on the directly to the cerium coordinated water molecules could have been located in the electron density map and have been refined with free U_iso_(H). The lattice water molecules in **2** (O21, O21A, O22, O22A, O23, O23A) and the carbon atoms (C12 and C14) in the acetone solvent molecule in **4** have been refined without hydrogen atoms, due to their disorder and the resulting larger atomic displacement parameters. The crystal data and details on the structure refinement are given in Table 6 and further information in Table S5 (for **1**), Table S7 (for **2**), Table S11 (for **3**) and Table S14 (for **4**). Graphics were drawn with DIAMOND [39]. The CCDC numbers are 2573145–2573147 for **1**–**3**, respectively and 2573144 for **4** and contain supplementary crystallographic data reported in this paper. These data can be obtained free of charge from the Cambridge Crystallographic Data Centre (CCDC) via https://www.ccdc.cam.ac.uk/data_request/cif (accessed on 20 July 2026).

### 3.2. Synthesis of Tetrachlorido-bis(dimethoxyethane)cerium(IV), [CeCl_4_(DME)_2_] (***1***)

To a solution of 25 mL of thionylchloride in 50 mL DME, 5 g of finely ground Ce(OH)_4_ (24 mmol) is slowly added within 5 min. After stirring the dark red solution for 3 h, the red solid was isolated by vacuum filtration and washed with ice-cold acetone (5 × 3 mL). The solid was stored under inert atmosphere in a flask. Intensely red crystals (Figure S1) crystallized from the acetone washing solution over the next three hours upon evaporation from an open vessel. (Yield: 8.93 g, 80% based on Ce(OH)_4_).

### 3.3. Synthesis of Diaqua-bis{[η^5^-cyclopentadienyltris(dimethylphosphonato-1κP:2κO)cobaltate(III)]}cerium(III) Chloride 2.5 Hydrate, [Ce(L_OMe_)_2_(H_2_O)_2_]Cl·2.5H_2_O (***2***)

To a solution of 153.1 mg of **1** (0.736 mmol) in 70 mL of acetone, 169.5 mg of solid NaL_OMe_ (0.358 mmol) was added, and the resulting mixture was stirred for 4 h. After 4 days of evaporation from an open vial most of the solvent afforded intense green crystals (Figure S1). The crystals were washed three times with 3 mL of ice-cold water. (Yield: 134 mg, 79% based on L). ^1^H NMR (CDCl_3_). δ 3.82 (s, 36H, CH_3_), 5.13 (s, 10H, Cp) (Figure S5). ^31^P-{^1^H} NMR (CDCl_3_): δ 160.20 (Figure S7). Anal. calc. excluding lattice water, C_38_H_100_Ce_2_Cl_2_Co_4_O_40_P_12_ (**3**—5H_2_O): C, 21.2; H, 4.7. Found: C, 22.8; H, 4.5. Fully dehydrated, C_38_H_92_Ce_2_Cl_2_Co_4_O_36_P_12_ (**3**—9H_2_O): C, 21.9; H, 4.5. Found: C, 22.8; H, 4.5. FT-IR (KBr): 3434 cm^−1^ (ν(O–H)), 1628 cm^−1^ (δ(H–O–H)), 1121 cm^−1^ (ν_as_C–O–(P))), 1036 cm^−1^ (ν_s_(C–O–(P))), 1003 cm^−1^ (δ(CH) [Cp]), 775 cm^−1^ (ρ(CH_3_)), 586 cm^−1^ (δ(P=O)), more information in Section S10. Infrared spectra.

### 3.4. Synthesis of Triaqua-Chlorido-[η^5^-cyclopentadienyltris(dimethylphosphonato-1κP:2κO)cobaltate(III)]cerium(III) Chloride, [CeCl(L_OMe_)(H_2_O)_3_]Cl (***3***) and Dichlorido-bis{[η^5^-cyclopentadienyltris(dimethylphosphonato-1κP:2κO)cobaltate(III)]}cerium(IV) Acetone Solvate, [CeCl_2_(L_OMe_)_2_]·acetone (***4***)

To a solution of 81.26 mg of **1** (0.390 mmol) in 40 mL of acetone, 168.71 mg of solid NaL_OMe_ (0.356 mmol) were added and stirred for 4 h. After 2 days, once most of the solvent had evaporated from an open vial, and slightly yellowish crystals were obtained, together with trace amounts of tiny intense red crystals of **4**, the crystals were washed three times with 3 mL each of ice-cold water and only the slightly yellowish crystals remained (Figure S1). (Yield of **3**: 185.23 mg, 73% based on L. ^1^H NMR (CDCl_3_). δ 3.84 (s, 18H, CH_3_), 5.12 (s, 5H, Cp) (Figure S6). ^31^P-{^1^H} NMR (CDCl_3_): δ 159.43 (Figure S8). Anal. Calc. for C_11_H_29_CeCl_2_CoO_12_P_3_: C, 18.5; H, 4.1. Found: C, 18.6; H, 4.0. FT-IR (KBr): 3416 cm^−1^ (ν(O–H)), 1630 cm^−1^ (δ(H–O–H)), 1132 cm^−1^ (ν_as_(C–O–(P))), 1034 cm^−1^ (ν_s_(C–O–(P))), 1001 cm^−1^ (δ(CH) [Cp]), 779 cm^−1^ (ρ(CH_3_)), 588 cm^−1^ (δ(P=O)), more information in Section S10. Infrared spectra.

## 4. Conclusions

Compound **1** was shown to be a useful starting material for cerium complexes, exemplified here through the synthesis of Ce complexes with the Kläui ligand η^5^-cyclopentadienyltris(dimethylphosphonato)cobaltate(III), [Co(η^5^-C_5_H_5_){P(O)(OMe)_2_}_3_]^−^ (L_OMe_). Among the three obtained complexes [Ce(L_OMe_)_2_(H_2_O)_2_]Cl·2.5H_2_O (**2**) and [CeCl(L_OMe_)(H_2_O)_3_]Cl (**3**), and [CeCl_2_(L_OMe_)_2_]·C_3_H_6_O (**4**) complexes **3** and **4** also contain chlorido ligands which could be substituted further. The structures of **1**–**3** show distorted square antiprism shapes while **4** represents a distorted triangular dodecahedron for the cerium atoms with coordination number eight and a different number of O and Cl donor atoms. The polymeric structure of **3** also differs to the molecular structures of **2** and **3**, because of the 1-dimensional chain, which resulted through the bridging action of chlorine between the cerium atoms. This results also in the space group *P*4_2_*bc*, while **2** and **3** share the space group *P*2_1_2_1_2. It is noteworthy that all three space groups are acentric. Thereby, the complexes **2**-**4** complement the series of compounds containing the L_OEt_ Kläui ligand.

## Figures and Tables

**Table 1:** Bond lengths around cerium in **1** ^a^.

	Bond Length [Å]
Ce1–Cl1	2.5795(8)
Ce1–Cl2	2.6107(8)
Ce1–Cl3	2.6008(8)
Ce1–Cl4	2.6038(8)
Ce1–O1	2.603(2)
Ce1–O2	2.584(2)
Ce1–O3	2.547(2)
Ce1–O4	2.586(3)

^a^ Bond angles around Ce are given in Table S6.

**Table 5:** Oxidation states of Ce by the bond valence sum analysis (BVS) for **1**–**4** ^a^.

Complex	BVS for Ce with R_0_(Ce(III)–O) and R_0_(Ce(III)–Cl) ^a^	BVS for Ce with R_0_(Ce(IV)–O) and R_0_(Ce(IV)–Cl) ^a^
[CeCl_4_(DME)_2_] (**1**)	+4.23	+3.78
[Ce(L_OMe_)_2_(H_2_O)_2_]Cl·2.5H_2_O (**2**)	+3.05	+2.77
[CeCl(L_OMe_)(H_2_O)_3_]Cl (**3**)	+2.94	+2.66
[CeCl_2_(L_OMe_)_2_]·C_3_H_6_O (**4**)	+4.39	+3.96

^a^ R_0_(Ce(III)–O) = 2.112 Å [32], R_0_(Ce(III)–Cl) = 2.504 Å [33], R_0_ (Ce(IV)–O) = 2.076 Å [33], R_0_(Ce(IV)–Cl) = 2.46 Å [18]. The reported R_0_(Ce(IV)–Cl) values for the coordination number of eight represent approximations, as relevant parameters are not fully available in existing databases and the literature. The R_ij_ values are listed in Table 1, Table 2, Table 3 and Table 4 for **1**–**4**, respectively.

**Table 6:** Crystal and structure refinement data for **1**–**4**.

	1	2	3	4
Empirical formula	C_8_H_20_CeClO_4_	2[C_19_H_50_CeCo_2_O_20_P_6_] ·2Cl·5H_2_O	C_11_H_29_CeClCoO_12_P_3_·Cl	C_22_H_46_CeCl_2_Co_2_O_18_P_6_·C_3_H_6_O
M/g mol^−1^	462.16	2245.67	716.20	1171.32
T/K	150	150	150	150
Wavelength/Å	0.701073	1.54184	1.54184	1.54184
Crystal system	Monoclinic	Orthorhombic	Tetragonal	Orthorhombic
Space group	*P*2_1_/*c*	*P*2_1_2_1_2	*P*4_2_*bc*	*P*2_1_2_1_2
a/Å	16.5744(6)	20.9616(2)	21.89770(10)	20.8164(2)
b/Å	7.26589(16)	20.8611(2)	21.89770(10)	11.51080(10)
c/Å	14.8768(5)	10.34780(10)	9.95180(10)	9.1672010)
β/deg	115.735(4)	90	90	90
V/Å^3^	1613.87(8)	4524.91(8)	4771.98(6)	2196.58(4)
Z	4	2	8	2
Calcd. Density/g cm^−3^	1.902	1.641	1.994	1.762
μ/mm^−1^	3.48	16.50	24.46	17.51
F(000)/e	904	2252	2840	1168
Crystal size/mm^3^	0.28 × 0.1 × 0.09	0.46 × 0.29 × 0.25	0.96 × 0.11 × 0.07	0.16 × 0.1 × 0.07
θ Range/deg	2.7–36.5	3.0–79.2	2.8–78.0	4.2–76.6
Reflections collected	12133	40891	16828	8979
Independent reflections; R_int_	8235; 0.076	9252; 0.086	4420; 0.043	4071; 0.032
Completeness to θ_max_/%	1.000	0.999	1.000	0.998
*R*_1_; w*R*_2_ (I > 2 σ(I)) ^1^	0.0509; 0.1185	0.0692; 0.1883	0.0302; 0.0812	0.0363; 0.0964
*R*_1_; w*R*_2_ (all data) ^2^	0.0692; 0.1277	0.0696; 0.1890	0.0310; 0.0817	0.0371; 0.0970
Goodness-of-fit ^3^	0.97	1.07	1.09	1.07
∆ρfin (max; min)/e Å^−3 4^	2.34; −1.56	1.30; −1.32	0.98; −1.36	0.83; −1.05
Flack parameter	-	0.003(6)	−0.010(4)	−0.011(3)
CCDC no.	2573145	2573146	2573147	2573144

^1^ *R*_1_ = ∑ | |F_o_| − |F_c_| |/∑|F_o_|. ^2^ w*R*_2_ [∑*w*(F_o_^2^ − F_c_^2^)^2^/∑[w(F_o_^2^)^2^]^1/2^, w = [σ^2^(F_o_^2^) + (*a*P)^2^ + *b*P]^−1^, where P = (F_o_^2^ + 2F_c_^2^)/3, *a* and *b* are constants adjusted by the program. ^3^ GoF = S = [∑*w*(F_o_^2^ − F_c_^2^)^2^/(n_obs_ − n_param_)]^1/2^, where n_obs_ is the number of data and n_param_ the number of refined parameters. ^4^ Largest peak and hole in final difference map.

## Data Availability

The original contributions presented in this study are included in the article/Supplementary Material. Further inquiries can be directed to the corresponding author(s). Crystallographic data for compounds **1**–**4** have been deposited at the CCDC under CCDC-2573145, CCDC-2573146, CCDC-2573147, and CCDC-2573144, respectively. These data can be obtained free of charge from the Cambridge Crystallographic Data Center via https://www.ccdc.cam.ac.uk/data_request/cif (accessed on 20 July 2026).

## Supplementary Materials

The following supporting information can be downloaded at: https://www.mdpi.com/article/10.3390/molecules31162865/s1, Section S1: List of chemicals; Section S2: Crystal images and powder X-ray diffraction; Section S3: ^1^H-, and ^31^P-NMR spectra of [Ce(L_OMe_)_2_(H_2_O)_2_]Cl·2.5H_2_O (**2**) and 1D-[Ce(μ-Cl)(L_OMe_)(H_2_O)_3_]Cl (**3**); Section S4: Continuous shape measure calculations for complexes [CeCl_4_(DME)_2_] (**1**), [Ce(L_OMe_)_2_(H_2_O)_2_]Cl·2.5H_2_O (**2**), 1D-[Ce(μ-Cl)(L_OMe_)(H_2_O)_3_]Cl (**3**) and [CeCl_2_(L_OMe_)_2_]·acetone (**4**) and the compared literature structures, with use of SHAPE V2.1 software; Section S5: X-ray structure of monoclinic [CeCl_4_(DME)_2_] (**1**); Section S6: X-ray structure of orthorhomic [Ce(L_OMe_)_2_(H_2_O)_2_]Cl·2.5H_2_O (**2**); Section S7: X-ray structure of tetragonal 1D-[Ce(μ-Cl)(L_OMe_)(H_2_O)_3_]Cl (**3**); Section S8: X-ray structure of orthorhombic [CeCl_2_(L_OMe_)_2_]·acetone (**4**); Section S9: Coordination polyhedra of cerium in discussed literature complexes; Section S10: Infrared spectra; Section S11: References. References [40,41] are cited in the Supplementary Materials.
