# Supplementary material for: Introducing Chlorido Cerium Complexes with the Methoxy Kläui Ligand [Co(η5-C5H5){P(O)(OMe)2}3]−
Source: Molecules. 2026 Aug 17;31(16):2865. doi: 10.3390/molecules31162865 (PMC13515825; doi:10.3390/molecules31162865)
Supplement: Supplementary file 1 [file molecules-31-02865-s001.zip › molecules-4480501-supplementary.pdf]

Supplementary Materials

Introducing Chlorido Cerium Complexes with the Methoxy Kläui Ligand $[Co(\eta^5\text{-}C_5H_5)\{P(O)(OMe)_2\}_3]^-$

Peter Ferber, Christoph Janiak *

Institut für Anorganische Chemie und Strukturchemie.
Heinrich-Heine-Universität Düsseldorf
40204 Düsseldorf, Germany
E-Mail: Christoph Janiak* - janiak@uni-duesseldorf.de * Corresponding author Fax: +49-211-81-12287; Tel: +49-211-81-12286
E-mail: Peter Ferber – peter.ferber@uni-duesseldorf.de

Table of Contents

Section S1. List of chemicals 2

Section S2. Crystal images and powder X-ray diffraction 3

Section S3. ^{1}H-, and ^{31}P-NMR spectra of $[Ce(L_{OMe})_2(H_2O)_2]Cl\cdot 2.5H_2O$ (**2**) and 1D-$[Ce(\mu\text{-}Cl)(L_{OMe})(H_2O)_3]Cl$ (**3**) 7

Section S4. Continuous shape measure calculations for complexes $[CeCl_4(DME)_2]$ (**1**), $[Ce(L_{OMe})_2(H_2O)_2]Cl\cdot 2.5H_2O$ (**2**), 1D-$[Ce(\mu\text{-}Cl)(L_{OMe})(H_2O)_3]Cl$ (**3**) and $[CeCl_2(L_{OMe})_2]$·acetone (**4**) and the compared literature structures, with use of the SHAPE V2.1 software [] 11

Sextion S5. X-ray structure of $[CeCl_4(DME)_2]$ (**1**) 14

Section S6. X-ray structure of $[Ce(L_{OMe})_2(H_2O)_2]Cl\cdot 2.5H_2O$ (**2**) 17

Section S7. X-ray structure of 1D-$[Ce(\mu\text{-}Cl)(L_{OMe})(H_2O)_3]Cl$ (**3**) 24

Section S8. X-ray structure of $[CeCl_2(L_{OMe})_2]$·acetone (**4**) 28

Section S9. Coordination polyhedra of cerium in discussed literature complexes. 31

Section S10. Infrared spectra 35

Section S11. References 36

Section S1. List of chemicals

Table S1. Overview of chemicals used.

**Chemical**	**Supplier**	**Purity**
Cerium(IV) hydroxide	Sigma-Aldrich	99.9%
NaL_{OMe} ($NaC_5H_5Co[P(O)(OMe)_2]_3$)	Synthesized according to the literature [1]	-
Acetone	Sigma Aldrich	99.8%
Dimethoxyethane	Grüssing GmbH	99%
Thionyl chloride	Acros Organics	99.7%

Section S2. Crystal images and powder X-ray diffraction

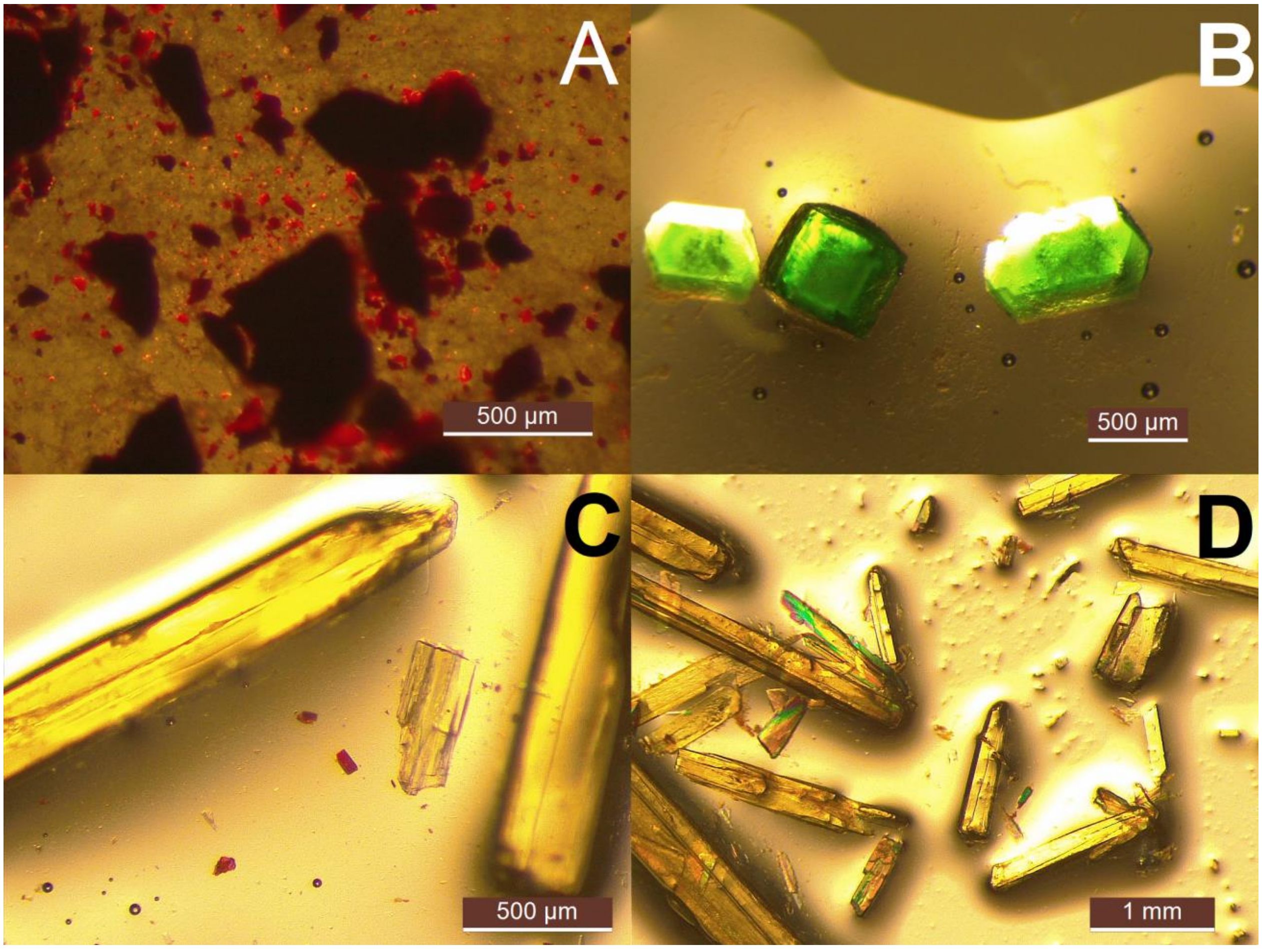

Figure S1. Optical micrograph of intensely red crystals of **1** (A, 60x magnification), intensely green crystals of **2** (B, 60x magnification), pale yellowish crystals of **3** and tiny intense red crystals of **4** (C, 60x magnification) and pale yellowish crystals of **3** after washing the sample (D, 25x magnification).

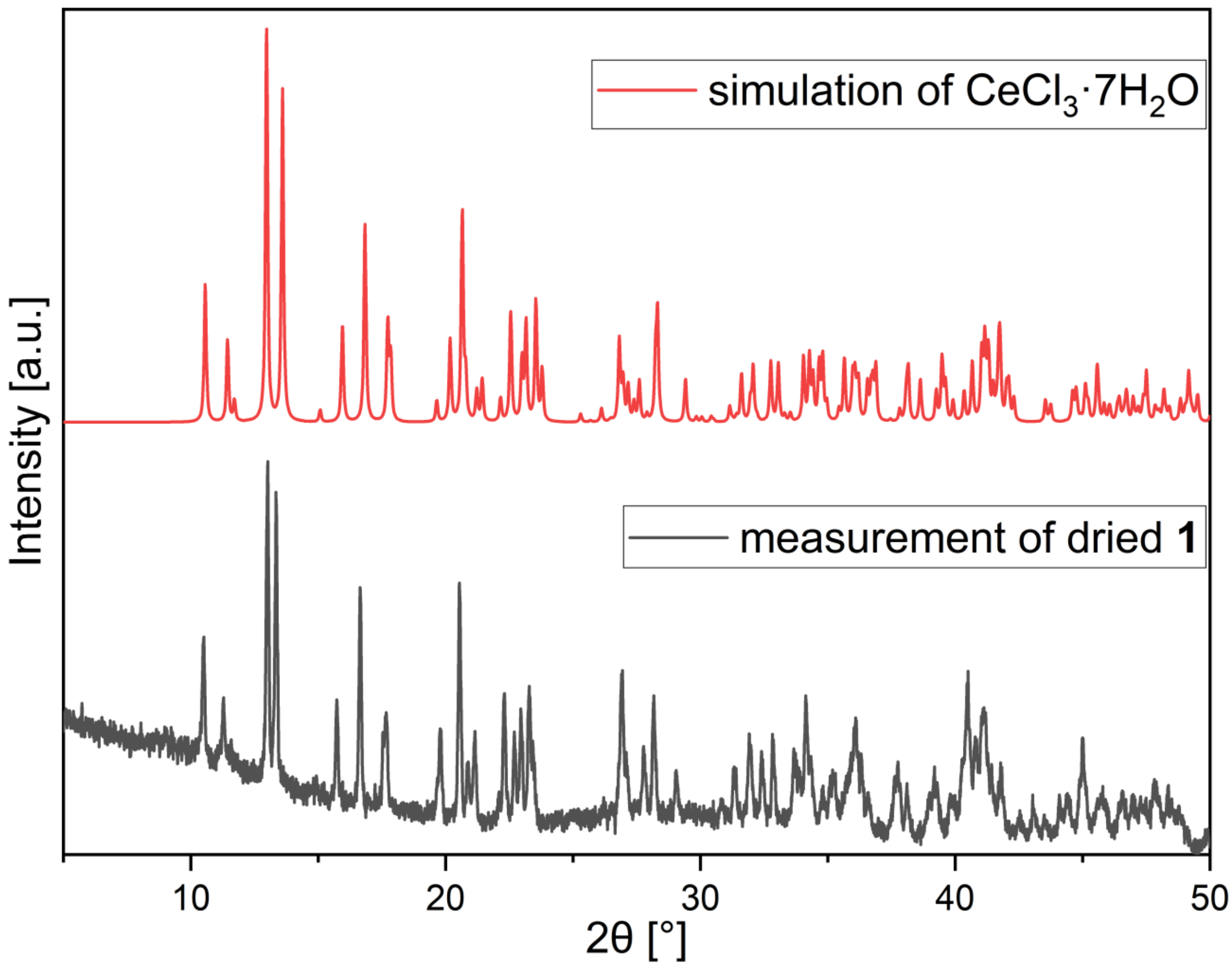

Figure S2. Powder X-ray diffraction (PXRD) pattern of the colorless sample yielded by drying of **1** in the vacuum oven (experimental, black) compared with the pattern simulated from single-crystal X-ray diffraction (SCXRD) data of $CeCl_3·7H_2O$ [COD ID: 1573329, Available online: file:///home/coder/svn-repositories/_RELOADED_COD/cod-reloaded/cif/1/57/33/1573329.cif] (red).

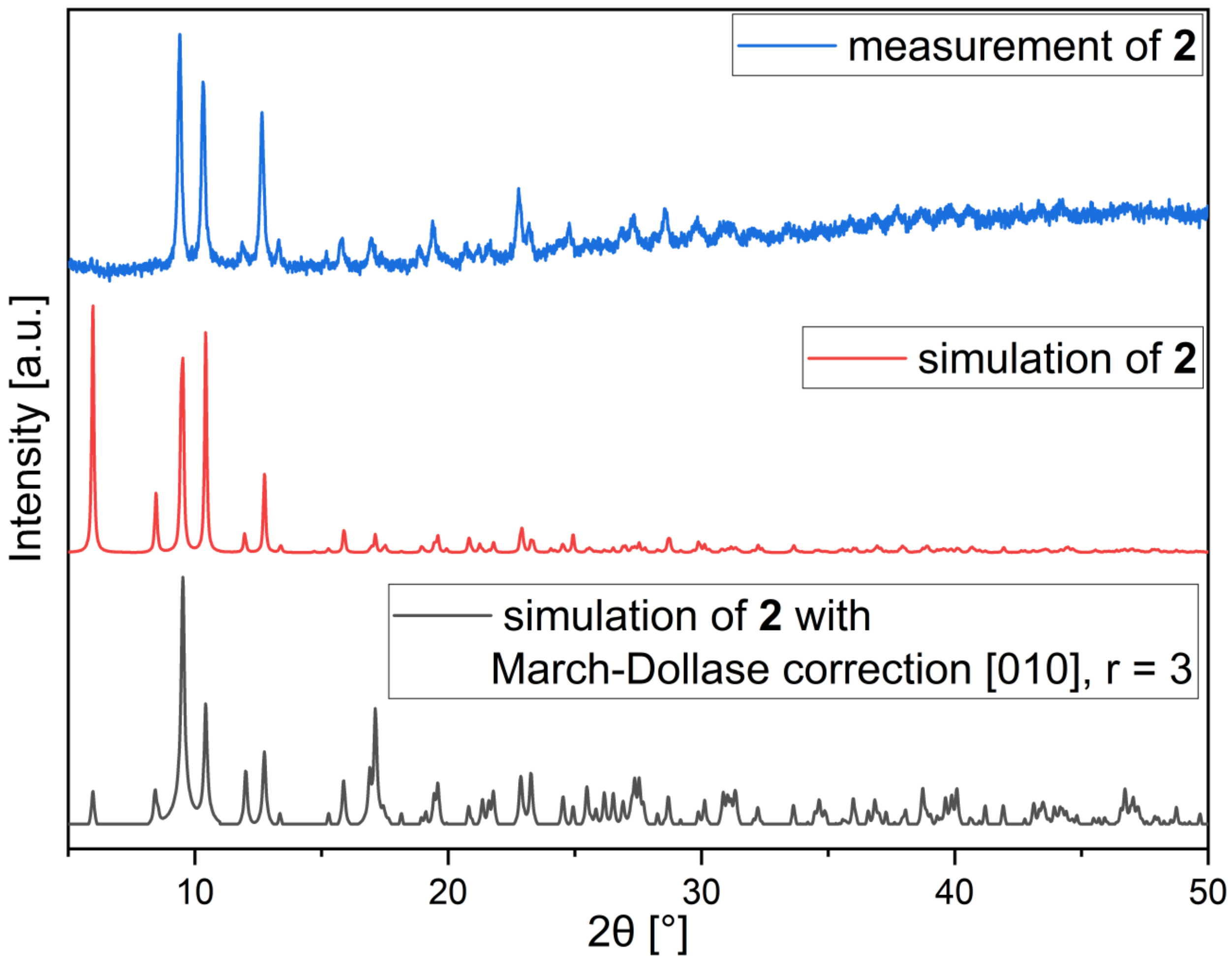

Figure S3. Powder X-ray diffraction (PXRD) pattern of **2** (experimental, blue) compared with the patterns simulated from single-crystal X-ray diffraction (SCXRD) data (red and black with March-Dollase correction [101], r = 3).

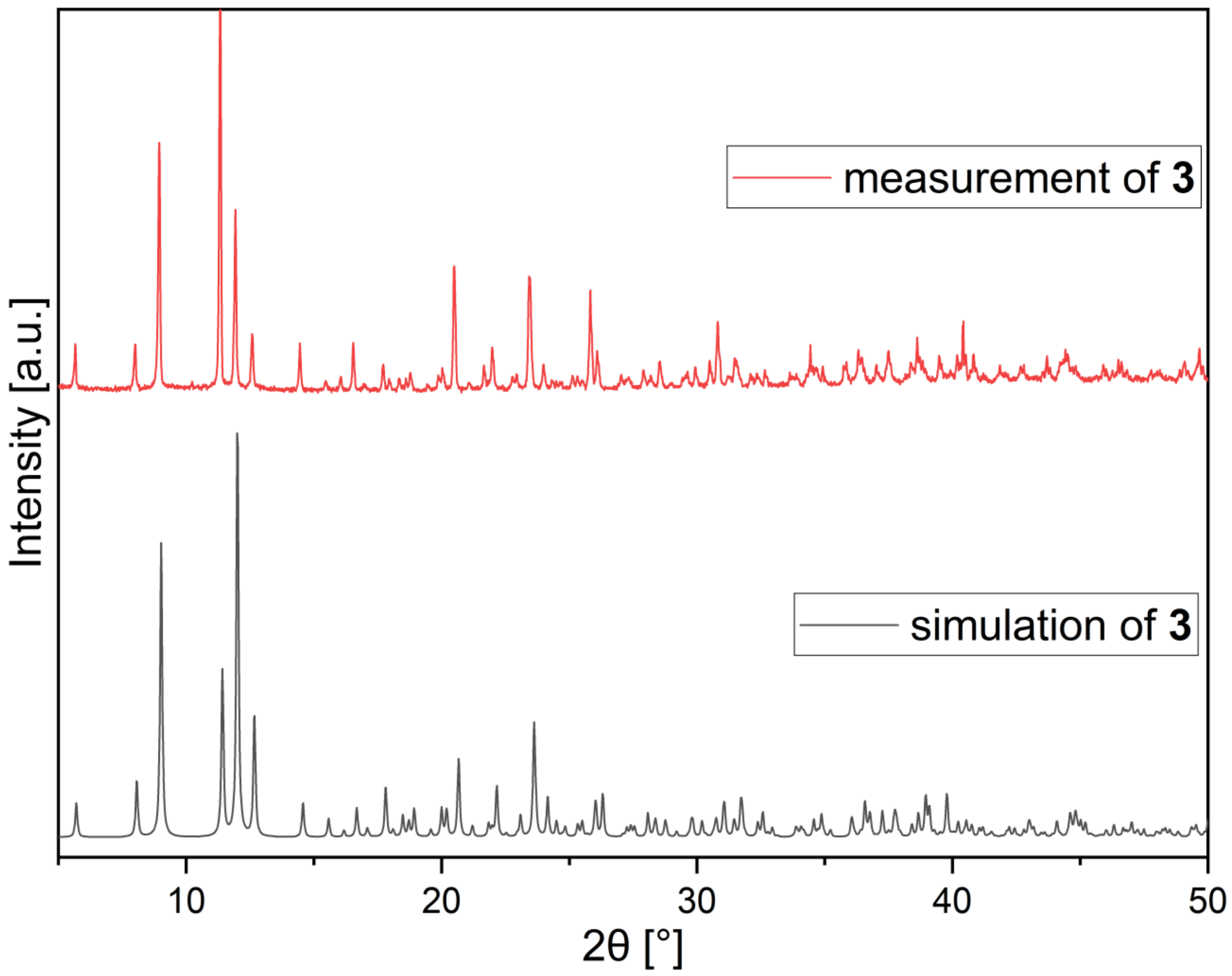

Figure S4. Powder X-ray diffraction (PXRD) pattern of **3** (experimental, red) compared with the pattern simulated from single-crystal X-ray diffraction (SCXRD) data (black).

Section S3. ^{1}H-, and ^{31}P-NMR spectra of $[Ce(L_{OMe})_2(H_2O)_2]Cl·2.5H_2O$ (**2**) and 1D-$[Ce(\mu\text{-}Cl)(L_{OMe})(H_2O)_3]Cl$ (**3**)

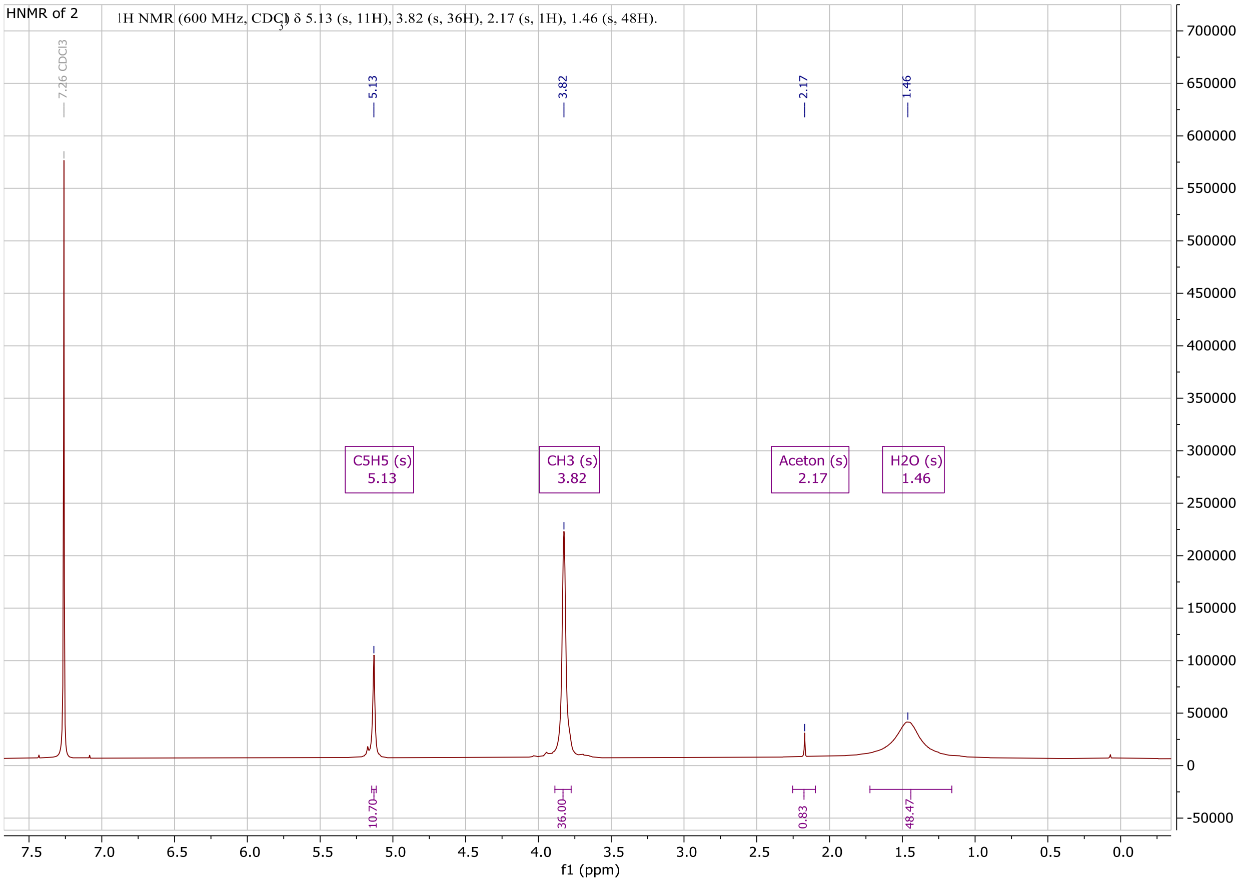

Figure S5. ^{1}H NMR spectrum of **2** in $CDCl_3$ (600 MHz).

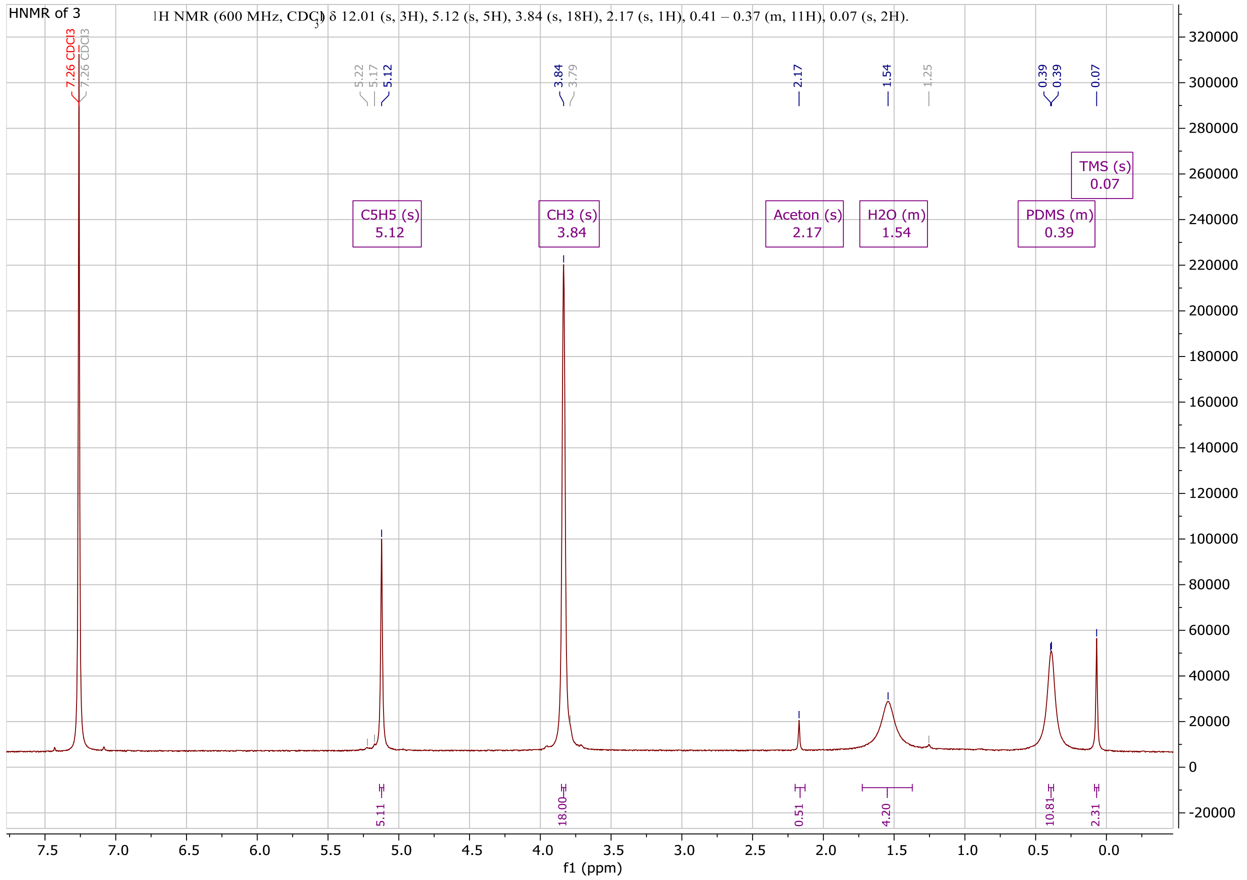

Figure S6. ^{1}H NMR spectrum of **3** in $CDCl_3$ (600 MHz). PDMS = polydimethylsiloxane, TMS = tetramethylsilane.

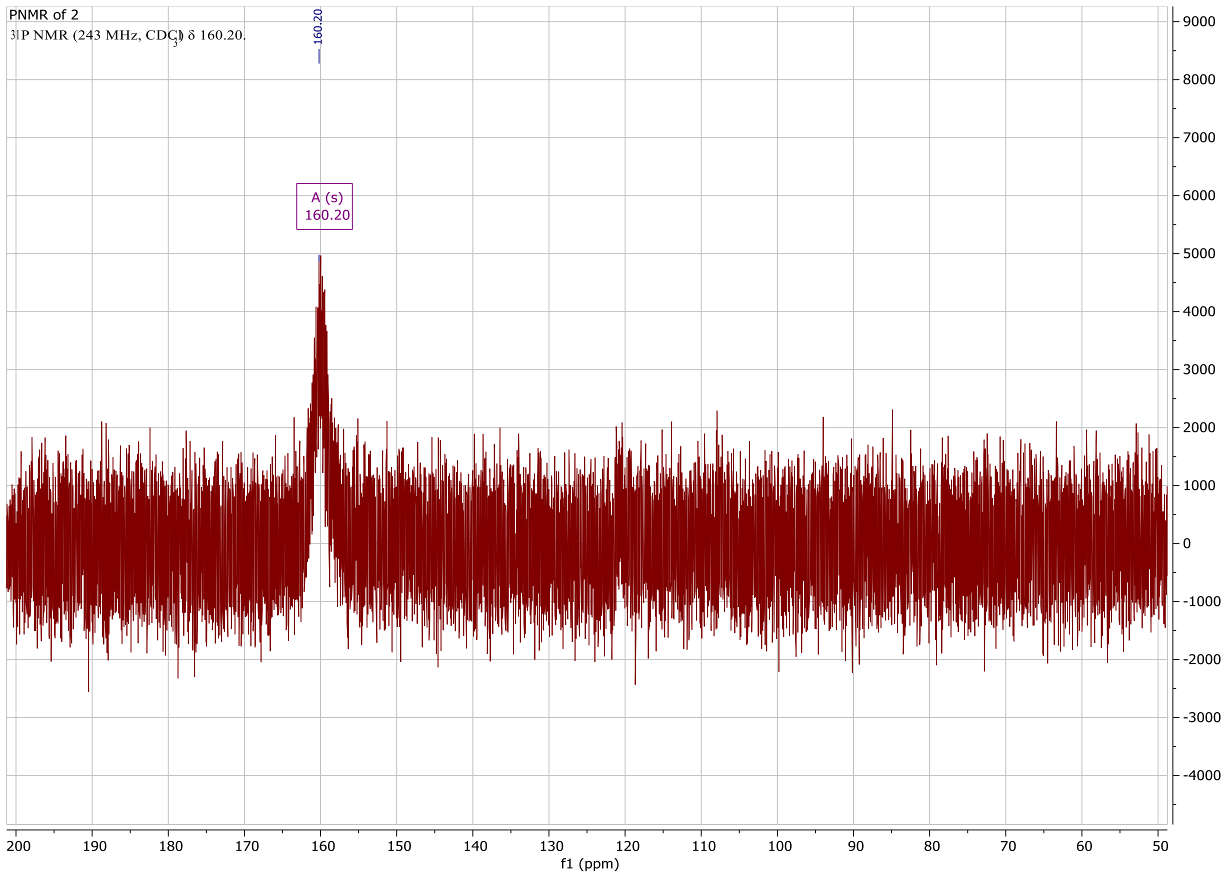

Figure S7. ^{31}P NMR spectrum of **2** in $CDCl_3$ (243 MHz).

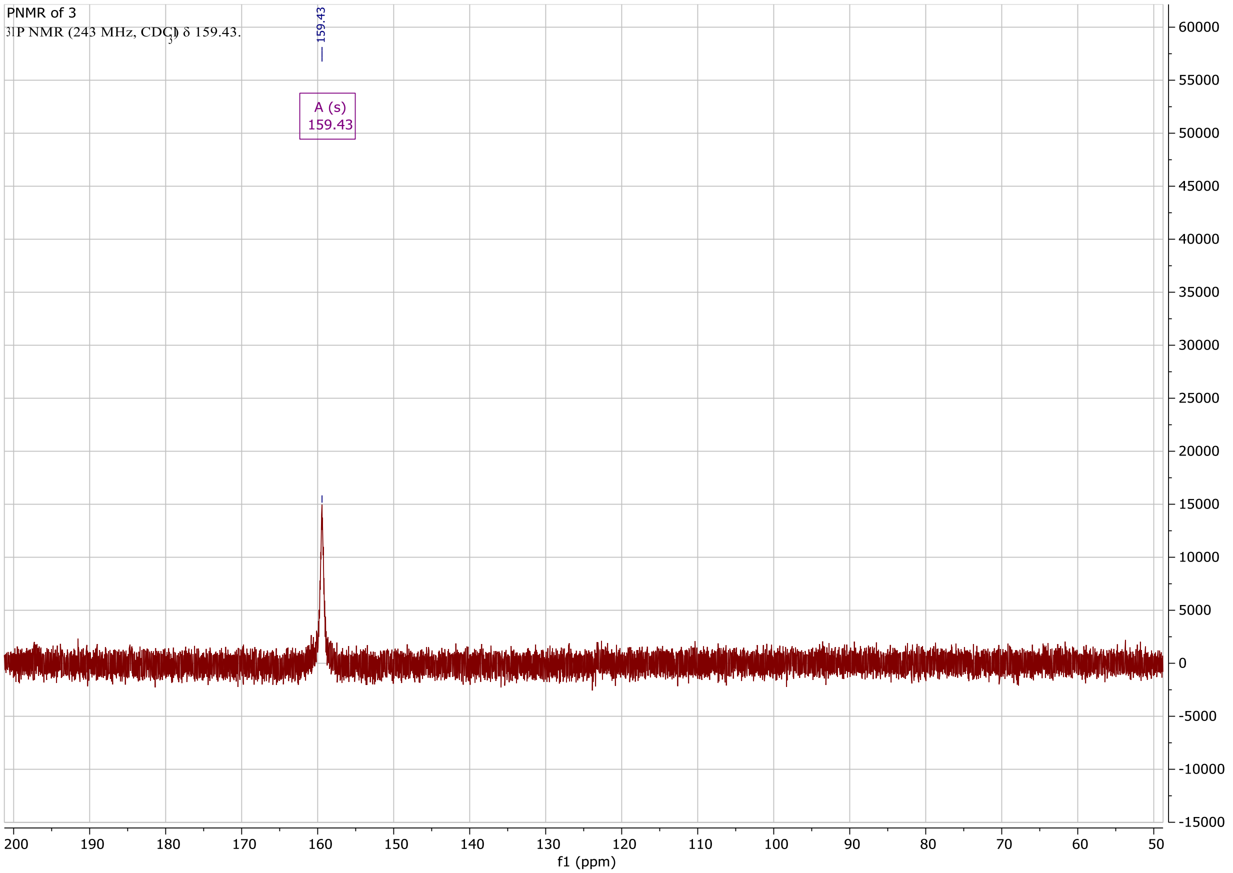

Figure S8. ^{31}P NMR spectrum of **3** in $CDCl_3$ (243 MHz).

Section S4. Continuous shape measure calculations for complexes $[CeCl_4(DME)_2]$ (**1**), $[Ce(L_{OMe})_2(H_2O)_2]Cl·2.5H_2O$ (**2**), 1D-$[Ce(\mu\text{-}Cl)(L_{OMe})(H_2O)_3]Cl$ (**3**) and $[CeCl_2(L_{OMe})_2]$·acetone (**4**) and the compared literature structures, with use of the SHAPE V2.1 software [2].

Table S2. Comparison of $[ML_8]$ geometries for complexes **1-4** using continuous shape measure (CShM) (calculated by Shape V2.1) [2]. [a]

**Geometries of $[ML_8]$**	**CShM (continuous shape measure)**			
	**1**	**2**	**3**	**4**
Octagon	30.062	29.148	27.889	32.67
Heptagonal pyramid	23.248	23.281	23.395	24.402
Hexagonal bipyramid	12.852	16.636	17.687	16.713
Cube	8.510	10.873	11.329	9.379
Square antiprism	**<u>1.370</u>**	**<u>0.653</u>**	**<u>1.101</u>**	2.767
Triangular dodecahedron	**1.979**	**1.928**	2.867	**<u>0.668</u>**
Johnson gyrobifastigium J26	13.068	15.243	13.352	16.252
Johnson elongated triangular bipyramid J14	28.615	27.374	26.377	30.298
Biaugmented trigonal prism J50	2.364	2.072	2.191	4.054
Biaugmented trigonal prism	**1.709**	**1.445**	**1.867**	2.922
Snub diphenoid	3.831	4.142	3.677	4.431
Triakis tetrahedron	9.315	11.484	11.142	9.526
Elongated trigonal bipyramid	24.772	23.092	22.495	25.19

[a] The lowest CShM values are underlined and shown in bold. Other low discussed CShM values are shown in bold.

Table S3. Comparison of geometric transitions for complexes **1-4** using continuous shape measure (CShM) (calculated by Shape V2.1) [2]

	**1**	**2**	**3**	**4**
Generalized Coordinate (TDD→SAPR)	83.2%	82.1%	100.3%	48.3%
Generalized Coordinate (SAPR→TDD)	69.2%	47.7%	62.0%	98.6%
Deviation from path (SAPR,TDD)	52.4%	29.8%	62.3%	46.8%
Generalized Coordinate (BTPR→SAPR)	86.7%	79.7%	90.7%	
Generalized Coordinate (SAPR→BTPR)	77.6%	53.5%	69.6%	
Deviation from path (SAPR,BTPR)	64.4%	33.2%	60.2%	

Table S4. Comparison of $[ML_8]$ geometries for complexes of the literature using continuous shape measure (CShM) (calculated by Shape V2.1). [a]

Geometries of [ML8]	CShM (continuous shape measure)			
	$[Ce_2Cl_6(DME)_4]$ [3]	$(N(CH_3)_4)_2$ $[CeCl_4(NO_3)_2]$ [4]	$[Ce(L_{OEt})_2(H_2O)_2]$ $(OTf)_2{\cdot}H_2O$ [5]	$[Ce(L_{OEt})_2(H_2O)_2]$ $(BF_4)_2$ [6]
Octagon	32.033	35.045	28.245	29.050
Heptagonal pyramid	22.737	27.907	23.790	23.284
Hexagonal bipyramid	12.392	6.395	16.720	17.450
Cube	7.081	13.875	9.825	10.302
Square antiprism	3.697	15.908	**0.238**	**0.244**
Triangular dodecahedron	**0.911**	13.969	2.338	2.710
Johnson gyrobifastigium J26	15.597	**4.237**	16.019	16.122
Johnson elongated triangular bipyramid J14	28.199	28.167	28.358	28.617
Biaugmented trigonal prism J50	3.983	14.811	2.218	2.671
Biaugmented trigonal prism	2.703	13.751	1.973	2.439
Snub diphenoid	3.935	13.680	4.507	4.874
Triakis tetrahedron	7.884	14.664	10.286	10.698
Elongated trigonal bipyramid	24.631	22.239	23.989	23.720

[a] The lowest CShM values are underlined and shown in bold.

Geometries of [ML8]	CShM (continuous shape measure)			
	$[CeCl(L_{OEt})_2$ $(OC_6H_3Cl_3\text{-}2,4,6)]$ [6]	$[CeCl(L_{OEt})_2$ $(OC_6H_3Cl_2\text{-}2,4)]$ [6]	$[CeCl_2(L_{OEt})_2]$ [7]	$[Ce(L_{OEt})_2(H_2O)_2]$ $[L_{OEt}]$ [5]
Octagon	30.250	30.454	33.193	28.33
Heptagonal pyramid	23.005	23.474	24.192	21.222
Hexagonal bipyramid	15.972	16.806	16.175	15.836
Cube	10.963	10.026	9.118	11.564
Square antiprism	**0.951**	1.234	3.413	2.029

Triangular dodecahedron	1.586	<u>**1.105**</u>	<u>**0.621**</u>	1.614
Johnson gyrobifastigium J26	15.018	15.796	16.016	13.259
Johnson elongated triangular bipyramid J14	28.912	28.261	29.484	26.825
Biaugmented trigonal prism J50	2.699	2.623	3.577	2.498
Biaugmented trigonal prism	1.907	1.746	2.530	<u>**1.456**</u>
Snub diphenoid	4.405	4.203	4.365	3.785
Triakis tetrahedron	11.451	10.527	9.327	12.377
Elongated trigonal bipyramid	25.232	24.993	25.544	21.983

[a] The lowest CShM values are underlined and shown in bold.

Sextion S5. X-ray structure of [$CeCl_4(DME)_2$] (**1**)

Table S5. Crystal data and structure refinement details of [$CeCl_4(DME)_2$] (**1**).

Crystal Data

$C_8H_{20}CeCl_4O_4$	F(000) = 904
Mr = 462.16	Dx = 1.902 Mg m^{-3}
Monoclinic, $P2_1/c$	Mo $K\alpha$ radiation, λ = 0.71073 Å
a = 16.5744 (6) Å	Cell parameters from 12133 reflections
b = 7.2659 (2) Å	θ = 2.7–36.5°
c = 14.8768 (5) Å	μ = 3.48 mm^{-1}
β = 115.735 (4)°	T = 150 K
V = 1613.88 (10) Å^3	Block, clear red
Z = 4	0.28 × 0.1 × 0.09 mm

Data collection

XtaLAB Synergy, Dualflex, HyPix	8235 independent reflections
Radiation source: micro-focus sealed X-ray tube, PhotonJet (Mo) X-ray Source	6190 reflections with $I > 2\sigma(I)$
Mirror monochromator	R_{int} = 0.076
Detector resolution: 10.0000 pixels mm^{-1}	θ_{max} = 38.0°, θ_{min} = 2.7°
ω scans	h = −27-26
	k = −12-11
Absorption correction: gaussian	l = −24-24
CrysAlis PRO 1.171.41.90a (Rigaku Oxford Diffraction, 2020) Numerical absorption correction based on gaussian integration over a multifaceted crystal model Empirical absorption correction using spherical harmonics, implemented in SCALE3 ABSPACK scaling algorithm.	25865 measured reflections
T_{min} = 0.482, T_{max} = 0.730	

Refinement

Refinement on F^2	Hydrogen site location: inferred from neighbouring sites
Least-squares matrix: full	H-atom parameters constrained
R[$F^2 > 2\sigma(F^2)$] = 0.0692<br>R[F^2, all data] = 0.0509	$w = 1/[\sigma^2(F_o^2) + (0.0644P)^2]$
wR($F^2 > 2\sigma(F^2)$ = 0.1185<br>wR(F^2, all data) = 0.1277	where $P = (F_o^2 + 2F_c^2)/3$
S = 0.97	$(\Delta/\sigma)_{max}$ = 0.002
8235 reflections	$\Delta\rho_{max}$ = 2.34 e Å^{-3}
158 parameters	$\Delta\rho_{min}$ = −1.56 e Å^{-3}
0 restraints	

Table S6. Selected bond lengths (Å) and angles (°) for the coordination polyhedron around cerium of [$CeCl_4(DME)_2$] (**1**).

Upper plane angles (ideal 70.5; 109.5) [8]	
Cl1–Ce1–O1	71.60(6)
O4–Ce1–Cl4	73.46(6)
Cl1–Ce1–O4	74.03(6)
O1–Ce1–Cl4	76.71(6)
Cl1–Ce1–Cl4	101.53(3)
O4–Ce1–O1	128.09(9)
lower plane angles (ideal 70.5; 109.5) [8]	
O2–Ce1–Cl3	72.21(6)
O3–Ce1–Cl2	73.04(6)
O2–Ce1–Cl2	73.74(5)
O3–Ce1–Cl3	74.63(6)
Cl3–Ce1–Cl2	105.79(3)
O3–Ce1–O2	123.50(9)
adjacent-neighbor interplanar angles (Ideal 99.6) [8]	
O2–Ce1–O1	63.82(7)
O3–Ce1–O4	65.44(9)
O4–Ce1–Cl2	79.42(7)
Cl3–Ce1–O1	80.37(6)
O3–Ce1–Cl4	82.48(6)
Cl1–Ce1–O2	82.97(6)
Cl1–Ce1–Cl2	84.44(3)
Cl3–Ce1–Cl4	84.81(3)
Angle between planes (O1–Cl1–O4–Cl4 to O2–Cl2–O3–Cl3)	0.2 (0.1)
RMS = root mean square deviation of O1–Cl1–O4–Cl4	0.25 Å
RMS = root mean square deviation of O2–Cl2–O3–Cl3	0.18 Å

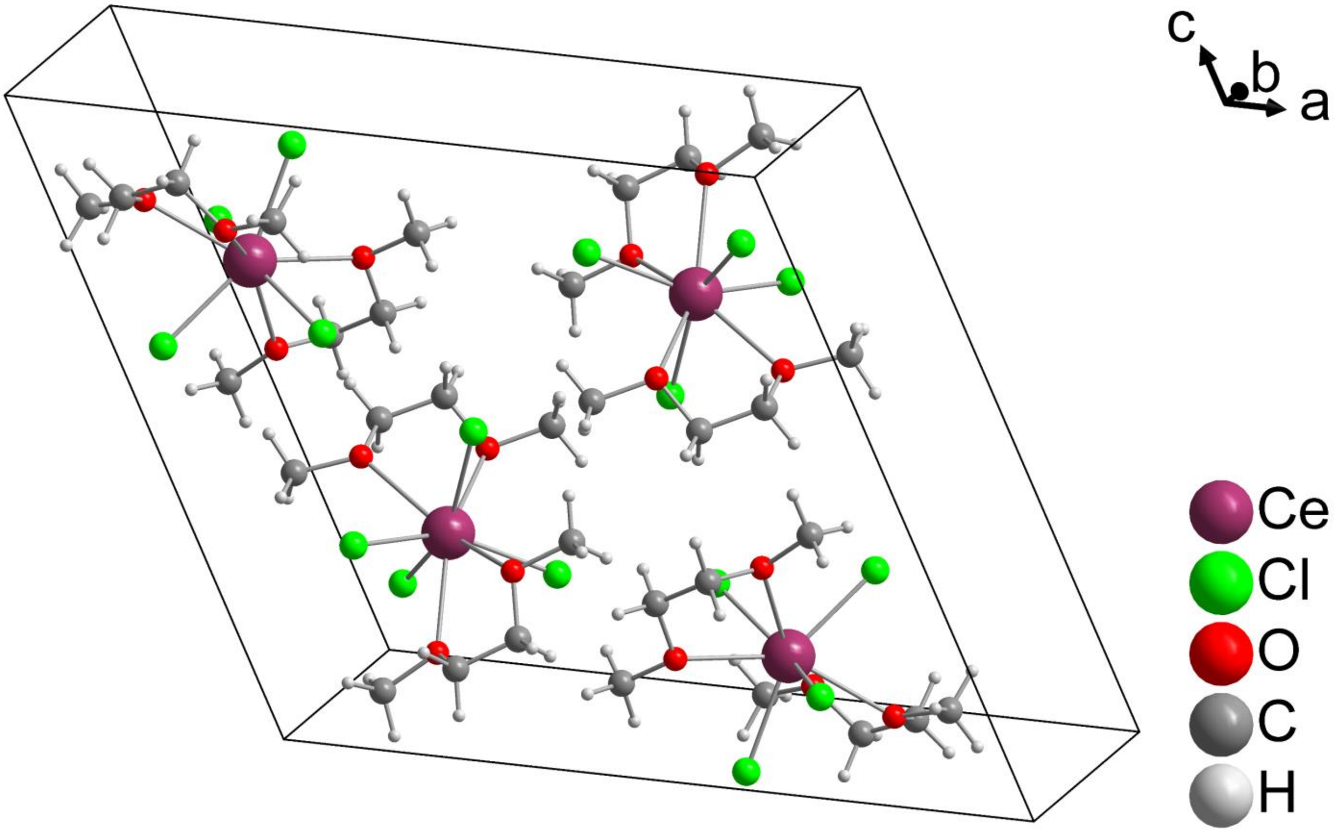

Figure S9. Packing diagram of $[CeCl_4(DME)_2]$ (**1**) with four asymmetric units.

Section S6. X-ray structure of [Ce(L_{OMe})$_2$(H_2O)$_2$]Cl·2.5H_2O (**2**)

Table S7. Crystal data and structure refinement details of [Ce(L_{OMe})$_2$(H_2O)$_2$]Cl·2.5H_2O (**2**).

Crystal data

2($C_{19}H_{50}CeCo_2O_{20}P_6$)·2(Cl)·5($H_2O$)	F(000) = 2252
M_r = 2245.67	D_x = 1.641 Mg m^{-3}
Orthorhombic, $P2_12_12$	Cu $K\alpha$ radiation, λ = 1.54184 Å
a = 20.9616 (2) Å	Cell parameters from 36444 reflections
b = 20.8611 (2) Å	θ = 3.0–77.7°
c = 10.3478 (1) Å	μ = 16.50 mm^{-1}
V = 4524.91 (8) Å^3	T = 150 K
Z = 2	Block, clear green
0.46 × 0.29 × 0.25 mm	

Data collection

XtaLAB Synergy, Dualflex, HyPix	9252 independent reflections
Radiation source: micro-focus sealed X-ray tube, PhotonJet (Cu) X-ray Source	9083 reflections with $I > 2\sigma(I)$
Mirror monochromator	R_{int} = 0.086
Detector resolution: 10.0000 pixels mm^{-1}	θ_{max} = 79.2°, θ_{min} = 3.0°
ω scans	h = −26-25
Absorption correction: gaussian	k = −25-24
CrysAlis PRO 1.171.41.90a (Rigaku Oxford Diffraction, 2020) Numerical absorption correction based on gaussian integration over a multifaceted crystal model Empirical absorption correction using spherical harmonics, implemented in SCALE3 ABSPACK scaling algorithm.	l = −13-12
T_{min} = 0.011, T_{max} = 0.390	

Refinement

Refinement on F^2	Hydrogen site location: mixed
Least-squares matrix: full	H atoms treated by a mixture of independent and constrained refinement
R[$F^2 > 2\sigma(F^2)$] = 0.0692<br>R[F^2, all data] = 0.0696	w = 1/[$\sigma^2(F_o^2) + (0.1475P)^2 + 4.4076P$]<br>where $P = (F_o^2 + 2F_c^2)/3$
wR($F^2 > 2\sigma(F^2)$) = 0.1884<br>wR(F^2, all data) = 0.1890	$(\Delta/\sigma)_{max}$ = 0.001
S = 1.07	$\Delta\rho_{max}$ = 1.30 e Å^{-3}
9252 reflections	$\Delta\rho_{min}$ = −1.32 e Å^{-3}
615 parameters	Absolute structure: Flack x determined using 3740 quotients [(I+)-(I-)]/[(I+)+(I-)] (Parsons, Flack and Wagner, Acta Cryst. B69 (2013) 249-259).
109 restraints	Absolute structure parameter: 0.003 (6)

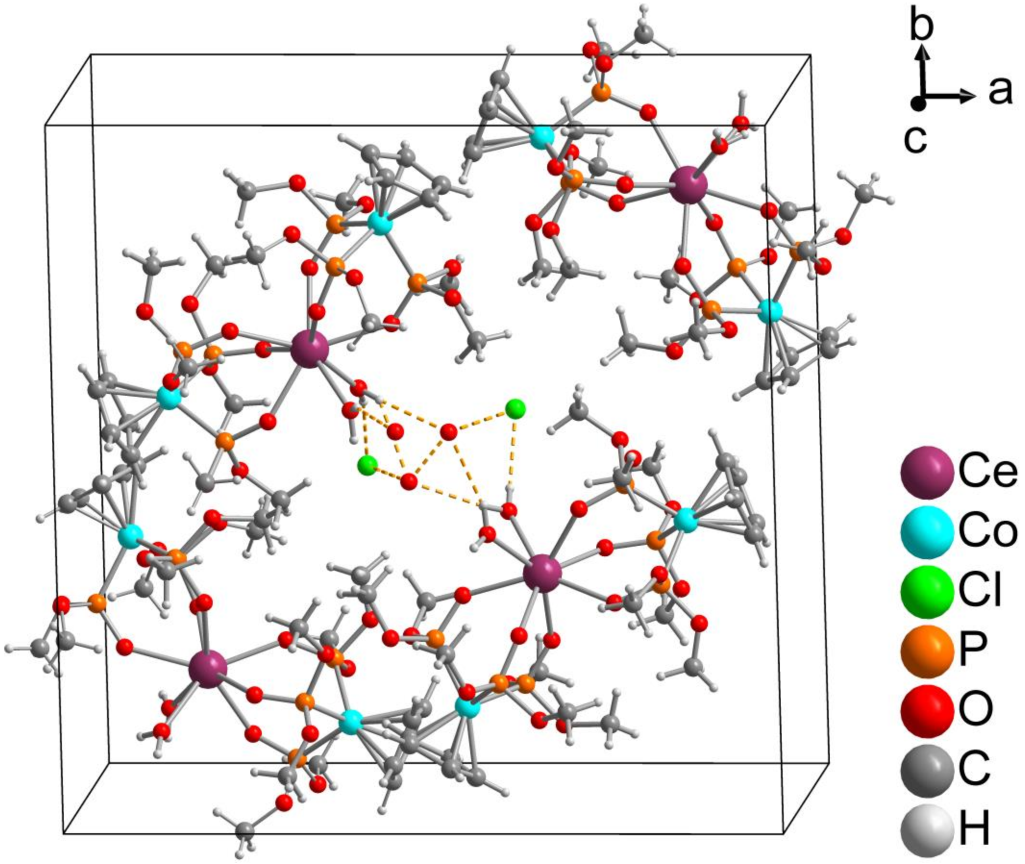

Figure S10. Packing diagram of $[Ce(L_{OMe})_2(H_2O)_2]Cl\cdot 2.5H_2O$ (**2**) with four asymmetric units. The dashed orange lines indicate possible hydrogen bonds from the water molecules to the chloride ions. The H atoms on the crystal water molecules could not be found (see also Figure S12 and Table S8).

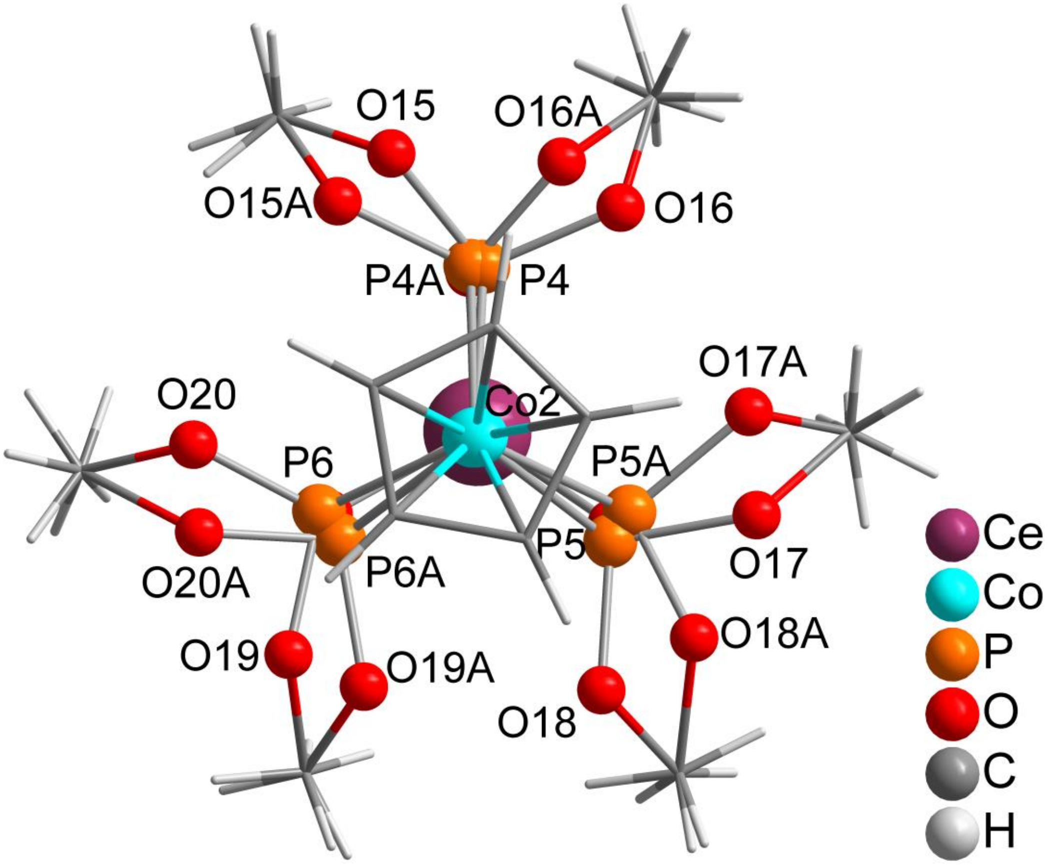

Figure S11. Disorder of the phosphonate groups in [Ce(L_{OMe})$_2$(H_2O)$_2$]Cl·2.5H_2O (**2**). (Ce, Co, O, and P in ball and stick model, C and H in capped stick model). The refined occupation factor for atoms A is 50%.

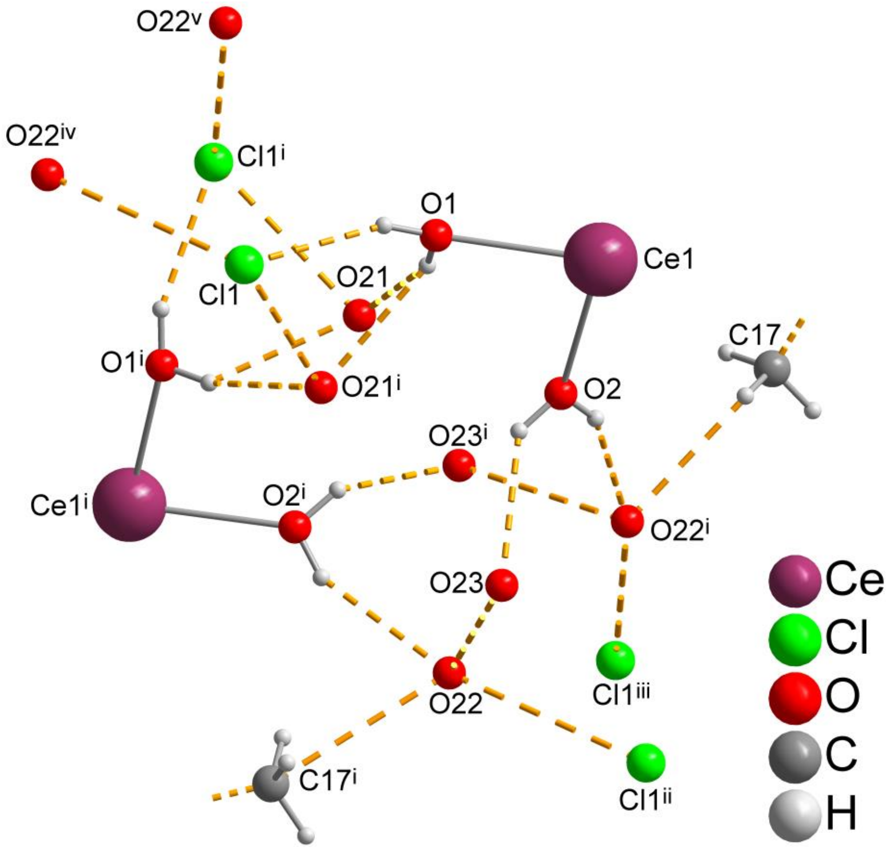

Figure S12. H-bonds of disorder part 1 of [Ce(L_{OMe})$_2$(H_2O)$_2$]Cl·2.5H_2O (**2**) with located hydrogen atoms on O1 and O2. The H atoms on the crystal water molecules (O21-O23) could not be found. Symmetry code: (i) −x+1, −y+1, z; (ii) x, y, z+1; (iii) -x+1, -y+1, z+1; (iv) x, y, +z-1; (v) -x+1, -y+1, z-1.

Table S8. Possible hydrogen bonds of part 1 of [Ce(L_{OMe})$_2$(H_2O)$_2$]Cl·2.5H_2O (**2**).

D—H···A [a]	D—H (Å)	H···A (Å)	D···A (Å)	D—H···A (°)
O1—H1*A*···O21	0.87	2.30	3.129 (13)	159
O1—H1*A*···O21^{i}	0.87	2.52	3.16 (5)	131 (4)
O1—H1*B*···Cl1	0.87	2.39	3.201 (15)	155 (3)
O2—H2*A*···O23	0.87	2.47	3.174 (1)	139 (11)
O2—H2*A*···O22^{i}	0.87	2.50	3.299 (18)	154 (4)
O21^{i}···Cl1	-	-	3.27 (7)	-
O22^{v}···Cl1	-	-	3.54 (3)	-
O23···O22	-	-	3.38 (3)	-
O22···C17^{i}	-	-	3.21 (3)	-

[a] The hydrogen atoms on O1 and O2, where located in the electron density map. The H atoms on the crystal water molecules (O21-O23) could not be found. Distances of O−O, O−C and O−Cl of oxygens without refined hydrogen atoms with calculated estimated standard deviations by from the SHELXL RTAB routine. Symmetry code: (i) −x+1, −y+1, z; (v) -x+1, -y+1, z-1.

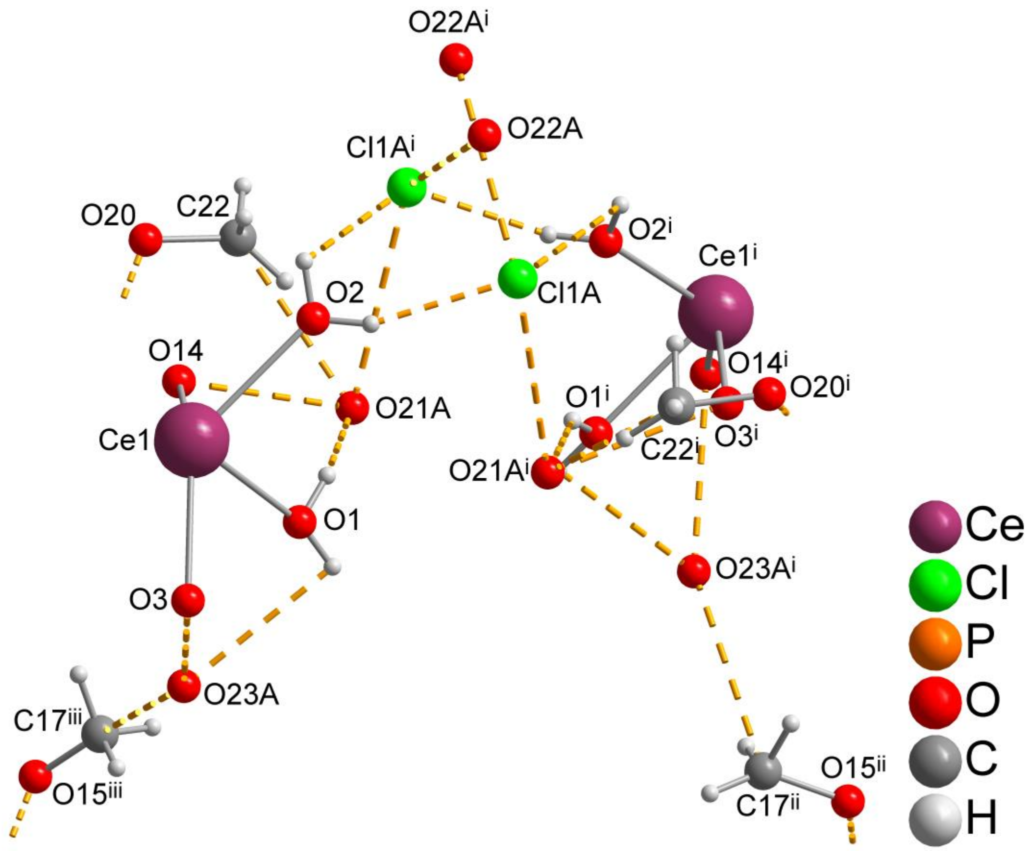

Figure S13. Hydrogen-bonds of disorder part 2 of $[Ce(L_{OMe})_2(H_2O)_2]Cl \cdot 2.5H_2O$ (**2**) with located hydrogen atoms on O1 and O2. The H atoms on the crystal water molecules (O21-O23) could not be found. Symmetry code: (i) −x+1, −y+1, z; (ii) -x+1, -y+1, z-1; (iii) x, y, -z-1.

Table S9. Possible hydrogen bonds in part 2 of $[Ce(L_{OMe})_2(H_2O)_2]Cl \cdot 2.5H_2O$ (**2**).

D—H···A [a]	D—H (Å)	H···A (Å)	D···A (Å)	D—H···A (°)
O1—H1*A*···O21*A*	0.87	2.06	2.80 (5)	142 (5)
O2—H2*A*···Cl1*A*	0.87	2.310	3.131 (17)	157 (4)
O23*A*···C17^{iii}	-	-	3.38 (3)	-
O21A^{i}···Cl1A	-	-	3.58 (7)	-
O22A^{i}···Cl1A	-	-	3.65 (4)	-
O21A···O14	-	-	2.93 (5)	-
O23A···O1	-	-	3.06 (3)	-
O23A···O3	-	-	3.020 (1)	-
O2···Cl1A^{i}	-	-	3.34 (3)	-

[a] The hydrogen atoms on O1 and O2, where located in the electron density map. The H atoms on the crystal water molecules (O21A-O23A) could not be found. Distances of O–O, O–C and O–Cl of oxygens without refined hydrogen atoms with calculated estimated standard deviations by from the SHELXL RTAB routine. Symmetry code: (i) −x+1, −y+1, z; (iii) x, y, -z-1.

Table S10. Selected bond lengths (Å) and angles (°) of [Ce(L_{OMe})$_2$(H_2O)$_2$]Cl·2.5H_2O (**2**).

Ce1—O1	2.546 (11)	P5A—O18A	1.592 (17)	P1—O7	1.623 (10)
Ce1—O2	2.583 (9)	P6—O14	1.502 (14)	P2—O4	1.488 (8)
Ce1—O3	2.479 (7)	P6—O19	1.591 (18)	P2—O8	1.590 (11)
Ce1—O4	2.447 (8)	P6—O20	1.565 (18)	P2—O9	1.620 (11)
Ce1—O5	2.419 (8)	P6A—O14	1.504 (14)	P3—O5	1.496 (8)
Ce1—O12	2.395 (8)	P6A—O19A	1.584 (19)	P3—O10	1.606 (9)
Ce1—O13	2.414 (7)	P6A—O20A	1.585 (18)	P3—O11	1.605 (9)
Ce1—O14	2.484 (7)	O1—H1A	0.8700 (14)	P4—O12	1.508 (14)
Co1—P1	2.162 (3)	O1—H1B	0.8700 (14)	P4—O15	1.608 (17)
Co1—P2	2.162 (3)	O2—H2A	0.8701 (15)	P4—O16	1.601 (18)
Co1—P3	2.160 (3)	O2—H2B	0.8701 (14)	P4A—O12	1.521 (11)
Co1—C1	2.109 (13)	O6—C6	1.39 (2)	P4A—O15A	1.634 (18)
Co1—C2	2.116 (13)	O7—C7	1.487 (16)	P4A—O16A	1.533 (15)
Co1—C3	2.099 (11)	O8—C8	1.34 (2)	P5—O13	1.520 (14)
Co1—C4	2.089 (11)	O9—C9	1.404 (18)	O15A—C17	1.33 (3)
Co1—C5	2.066 (11)	O10—C10	1.36 (2)	O16—C18	1.40 (2)
Co2—P4	2.162 (11)	O11—C11	1.451 (16)	O16A—C18	1.48 (2)
Co2—P4A	2.156 (9)	O15—C17	1.44 (3)	O17—C19	1.504 (17)
Co2—P5	2.159 (12)	Co2—C12	2.070 (11)	O17A—C19	1.21 (3)
Co2—P5A	2.167 (11)	Co2—C13	2.075 (11)	O18—C20	1.34 (2)
Co2—P6	2.166 (12)	Co2—C14	2.077 (12)	O18A—C20	1.63 (2)
Co2—P6A	2.160 (12)	Co2—C15	2.075 (10)	O19—C21	1.55 (2)
P5—O17	1.632 (18)	Co2—C16	2.083 (10)	O19A—C21	1.34 (3)
P5—O18	1.618 (17)	P1—O3	1.489 (8)	O20—C22	1.44 (2)
P5A—O13	1.525 (13)	P1—O6	1.593 (8)	O20A—C22	1.64 (3)
P5A—O17A	1.66 (2)				
O1—Ce1—O2	80.8 (4)	O6—P1—Co1	113.1 (4)	O13—P5A—O17A	108.8 (11)
O3—Ce1—O1	72.6 (4)	O6—P1—O7	99.2 (5)	O13—P5A—O18A	110.0 (9)
O3—Ce1—O2	136.0 (3)	O7—P1—Co1	105.2 (4)	O17A—P5A—Co2	103.6 (9)
O3—Ce1—O14	125.2 (3)	O4—P2—Co1	120.3 (3)	O18A—P5A—Co2	114.3 (9)
O4—Ce1—O1	143.5 (3)	O4—P2—O8	110.1 (5)	O18A—P5A—O17A	97.9 (13)
O4—Ce1—O2	114.9 (3)	O4—P2—O9	107.6 (5)	O14—P6—Co2	120.6 (8)
O4—Ce1—O3	73.9 (2)	O8—P2—Co1	109.8 (5)	O14—P6—O19	104.6 (10)
O4—Ce1—O14	144.4 (3)	O8—P2—O9	102.5 (8)	O14—P6—O20	112.2 (11)
O5—Ce1—O1	84.0 (4)	O9—P2—Co1	104.9 (4)	P4—Co2—P6	90.7 (5)
O5—Ce1—O2	68.1 (3)	O5—P3—Co1	119.9 (3)	P4A—Co2—P5A	90.2 (5)
O5—Ce1—O3	74.6 (2)	O5—P3—O10	110.6 (5)	P4A—Co2—P6A	94.9 (4)
O5—Ce1—O4	73.3 (3)	O5—P3—O11	107.7 (5)	P5—Co2—P4	94.2 (5)
O5—Ce1—O14	136.9 (3)	O10—P3—Co1	110.7 (4)	P5—Co2—P6	90.2 (5)
O12—Ce1—O1	141.0 (3)	O11—P3—Co1	106.4 (3)	P6A—Co2—P5A	89.8 (5)
O12—Ce1—O2	76.4 (3)	O11—P3—O10	99.2 (5)	O19—P6—Co2	110.1 (9)
O12—Ce1—O3	143.1 (3)	O12—P4—Co2	121.4 (8)	O20—P6—Co2	107.7 (9)

O12—Ce1—O4	75.4 (3)	O12—P4—O15	106.5 (10)	O20—P6—O19	99.6 (12)
O12—Ce1—O5	115.2 (3)	O12—P4—O16	107.6 (11)	O14—P6A—Co2	120.9 (8)
O12—Ce1—O13	77.8 (2)	O15—P4—Co2	114.3 (9)	O14—P6A—O19A	111.2 (11)
O12—Ce1—O14	73.6 (3)	O16—P4—Co2	104.0 (8)	O14—P6A—O20A	107.0 (11)
O13—Ce1—O1	104.7 (4)	O16—P4—O15	100.9 (13)	O19A—P6A—Co2	105.8 (11)
O13—Ce1—O2	143.8 (3)	O12—P4A—Co2	121.0 (5)	O19A—P6A—O20A	97.8 (15)
O13—Ce1—O3	78.0 (2)	O12—P4A—O15A	109.4 (10)	O20A—P6A—Co2	111.8 (10)
O13—Ce1—O4	82.2 (3)	O12—P4A—O16A	111.0 (10)	C18—O16—P4	116.1 (16)
O13—Ce1—O5	147.3 (2)	O15A—P4A—Co2	100.8 (7)	C18—O16A—P4A	122.4 (15)
O13—Ce1—O14	74.5 (3)	O16A—P4A—Co2	113.6 (9)	C19—O17—P5	124.0 (12)
O14—Ce1—O1	69.9 (4)	O16A—P4A—O15A	97.7 (12)	C19—O17A—P5A	117.8 (17)
O14—Ce1—O2	74.1 (3)	O13—P5—Co2	120.4 (8)	C20—O18—P5	117.9 (14)
P1—Co1—P2	89.14 (12)	O13—P5—O17	105.6 (9)	P5A—O18A—C20	120.3 (12)
P3—Co1—P1	92.73 (12)	O13—P5—O18	111.9 (10)	C21—O19—P6	124.8 (14)
P3—Co1—P2	90.66 (12)	O17—P5—Co2	111.3 (9)	C21—O19A—P6A	117.9 (19)
O3—P1—Co1	120.8 (3)	O18—P5—Co2	107.5 (8)	C22—O20—P6	115.8 (16)
O3—P1—O6	108.4 (5)	O18—P5—O17	97.9 (11)	P6A—O20A—C22	121.4 (16)
O3—P1—O7	107.8 (4)	O13—P5A—Co2	119.6 (7)		

Section S7. X-ray structure of 1D-[Ce(μ-Cl)(L_{OMe})(H_2O)$_3$]Cl (**3**)

Table S11. Crystal data an structure refinement details of 1D-[Ce(μ-Cl)(L_{OMe})(H_2O)$_3$]Cl (**3**).

Crystal data

$C_{11}H_{29}CeClCoO_{12}P_3$·Cl	D_x = 1.994 Mg m^{-3}
M_r = 716.20	Cu $K\alpha$ radiation, λ = 1.54184 Å
Tetragonal, $P4_2bc$	Cell parameters from 16828 reflections
a = 21.8977 (1) Å	θ = 2.8–78.0°
c = 9.9518 (1) Å	μ = 24.46 mm^{-1}
V = 4771.98 (6) Å^3	T = 150 K
Z = 8	Block, clear colourless
F(000) = 2840	0.96 × 0.11 × 0.07 mm

Data collection

XtaLAB Synergy, Dualflex, HyPix	4420 independent reflections
Radiation source: micro-focus sealed X-ray tube, PhotonJet (Cu) X-ray Source	
Mirror monochromator	4331 reflections with $I > 2\sigma(I)$
Detector resolution: 10.0000 pixels mm^{-1}	R_{int} = 0.043
ω scans	θ_{max} = 78.2°, θ_{min} = 2.9°
Absorption correction: gaussian	h = −26-25
CrysAlis PRO 1.171.41.90a (Rigaku Oxford Diffraction, 2020) Numerical absorption correction based on gaussian integration over a multifaceted crystal model Empirical absorption correction using spherical harmonics, implemented in SCALE3 ABSPACK scaling algorithm.	k = −24-27
T_{min} = 0.006, T_{max} = 0.729	l = −11-12
21451 measured reflections	

Refinement

Refinement on F^2	Hydrogen site location: mixed
Least-squares matrix: full	H atoms treated by a mixture of independent and constrained refinement
R[$F^2 > 2\sigma(F^2)$] = 0.0302<br>R[F^2, all data] = 0.0310	w = 1/[$\sigma^2(F_o^2)$ + (0.0523P)^2 + 7.6208P]<br>where $P = (F_o^2 + 2F_c^2)/3$
wR($F^2 > 2\sigma(F^2)$) = 0.1812<br>wR(F^2, all data) = 0.1817	$(\Delta/\sigma)_{max}$ = 0.001
S = 1.09	$\Delta\rho_{max}$ = 0.98 e Å^{-3}
4420 reflections	$\Delta\rho_{min}$ = −1.36 e Å^{-3}
271 parameters	Absolute structure: Flack x determined using 1783 quotients [(I+)-(I-)]/[(I+)+(I-)] (Parsons, Flack and Wagner, Acta Cryst. B69 (2013) 249-259).
5 restraints	Absolute structure parameter: −0.010 (4)

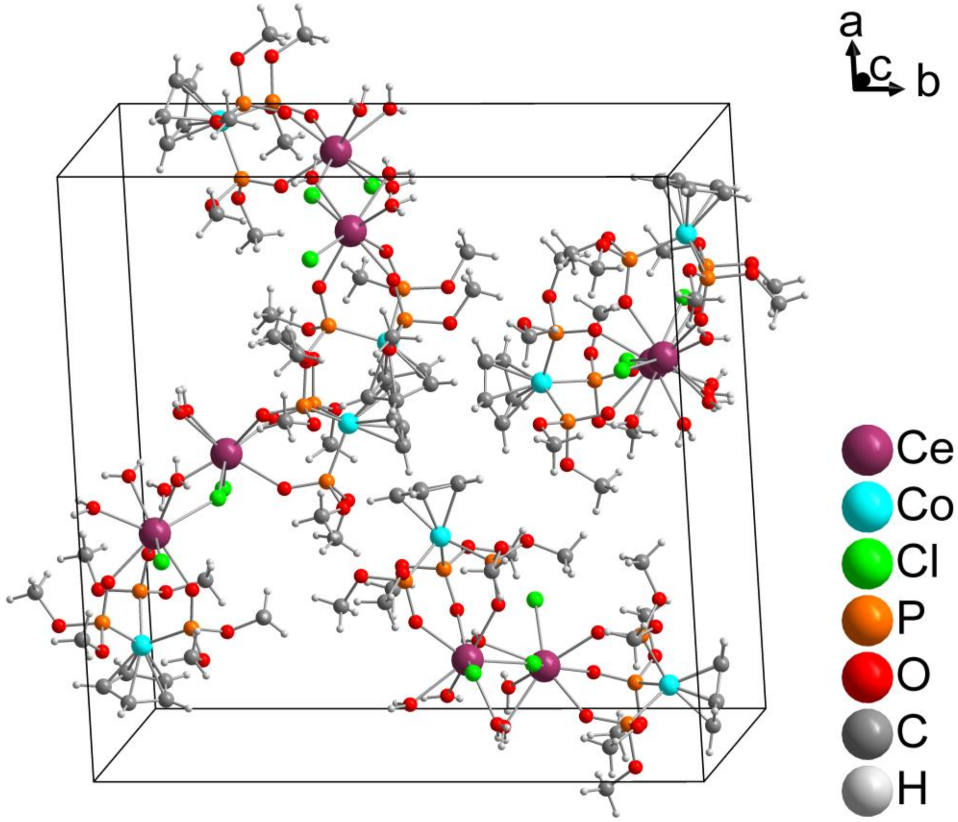

Figure S14. Unit cell of 1D-[Ce(μ-Cl)(L_{OMe})(H_2O)$_3$]Cl (**3**) with eight asymmetric units.

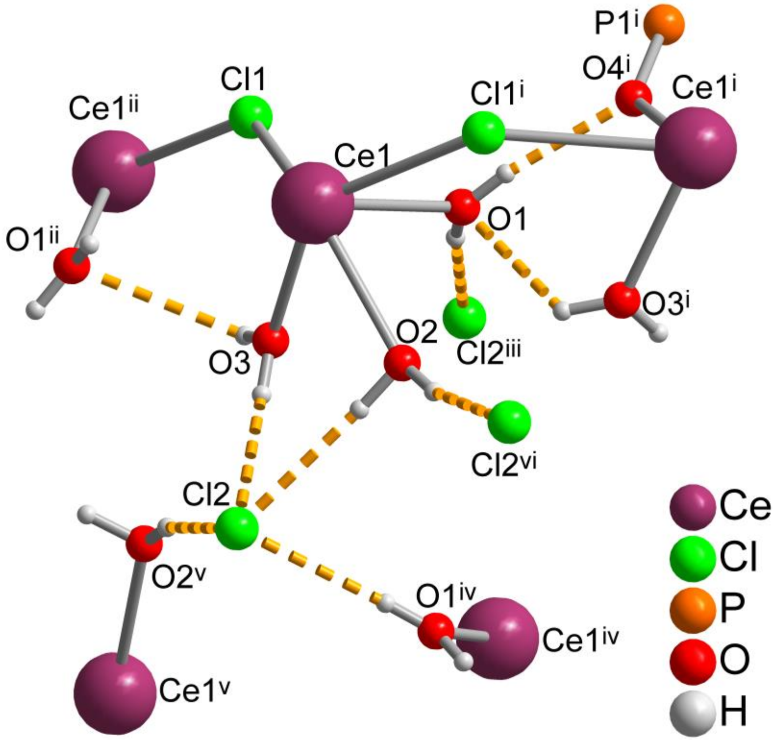

Figure S15. Hydrogen-bonds of 1D-[Ce(μ-Cl)(L_{OMe})(H_2O)$_3$]Cl (**3**). Symmetry codes: (i) y+1/2, x−1/2, z−1/2; (ii) y+1/2, x−1/2, z+1/2; (iii) x, y, z−1; (iv) −x+2, −y+1, z; (v) -y+3/2, -x+3/2, z+1/2; (vi) −y+3/2, −x+3/2, z−1/2.

Table S12. Hydrogen bonds in 1D-[Ce(μ-Cl)(LOMe)(H_2O)$_3$]Cl (**3**).

D—H···A	D—H (Å)	H···A (Å)	D···A (Å)	D—H···A (°)
O1—H1*A*···O4^{i}	0.77 (13)	2.07 (13)	2.831 (7)	172 (13)
O1^{iv} —H1*B*···Cl2	0.80 (12)	2.36 (12)	3.153 (6)	170 (11)
O2—H2*A*···Cl2^{vi}	0.91 (2)	2.25 (3)	3.138 (6)	164 (5)
O2—H2*B*···Cl2	0.88 (3)	2.38 (3)	3.257 (6)	176 (10)
O3—H3*A*···O1^{ii}	0.84 (12)	2.56 (12)	3.090 (8)	122 (9)
O3—H3*A*···O2^{ii}	0.84 (12)	2.36 (12)	3.149 (8)	157 (11)
O3—H3*B*···Cl2	0.82 (12)	2.27 (12)	3.082 (6)	171 (11)

Symmetry codes: (i) y+1/2, x−1/2, z−1/2; (ii) y+1/2, x−1/2, z+1/2; (iv) −x+2, −y+1, z; (vi) −y+3/2, −x+3/2, z−1/2.

Table S13. Selected bond lengths (Å) and angles (°) of 1D-[Ce(μ-Cl)(L_{OMe})(H_2O)$_3$]Cl (**3**).[a]

Ce1—Cl1	2.9137 (16)	P2—O9	1.598 (5)
Ce1—Cl1^{i}	2.9552 (18)	P2—O10	1.610 (5)
Ce1—O1	2.513 (5)	P3—O6	1.507 (5)
Ce1—O2	2.592 (5)	P3—O11	1.600 (5)
Ce1—O3	2.510 (5)	P3—O12	1.599 (5)
Ce1—O4	2.479 (5)	O1—H1A	0.77 (13)
Ce1—O5	2.370 (4)	O1—H1B	0.80 (12)
Ce1—O6	2.369 (4)	O2—H2A	0.91 (2)
Co1—P1	2.164 (2)	O2—H2B	0.88 (3)
Co1—P2	2.1729 (19)	O3—H3A	0.84 (12)
Co1—P3	2.166 (2)	O3—H3B	0.82 (12)
Co1—C1	2.074 (8)	O7—C6	1.452 (9)
Co1—C2	2.070 (8)	Cl1—Ce1—Cl1^{i}	123.33 (4)
Co1—C3	2.087 (7)	O1—Ce1—Cl1	74.87 (14)
Co1—C4	2.084 (7)	O1—Ce1—Cl1^{i}	70.70 (14)
Co1—C5	2.082 (8)	O1—Ce1—O2	75.47 (17)
Cl1—Ce1^{ii}	2.9552 (18)	O8—C7	1.453 (10)
P1—O4	1.507 (4)	O9—C8	1.452 (9)
P1—O7	1.593 (5)	O10—C9	1.433 (10)
P1—O8	1.607 (6)	O11—C10	1.445 (10)
P2—O5	1.507 (4)	O12—C11	1.445 (9)
O2—Ce1—Cl1^{i}	76.66 (13)	O4—P1—Co1	121.5 (2)
O2—Ce1—Cl1	134.48 (12)	O4—P1—O7	109.6 (3)
O3—Ce1—Cl1^{i}	138.42 (13)	O4—P1—O8	107.1 (3)
O3—Ce1—Cl1	72.33 (13)	O7—P1—Co1	104.9 (2)
O3—Ce1—O1	78.86 (18)	O7—P1—O8	100.0 (3)

O3—Ce1—O2	68.62 (18)	O8—P1—Co1	111.7 (2)
O4—Ce1—Cl1[i]	143.50 (11)	O5—P2—Co1	120.14 (18)
O4—Ce1—Cl1	73.77 (11)	O5—P2—O9	109.5 (3)
O4—Ce1—O1	143.87 (17)	O5—P2—O10	108.1 (3)
O4—Ce1—O2	116.08 (16)	O9—P2—Co1	105.8 (2)
O4—Ce1—O3	74.92 (16)	O9—P2—O10	99.4 (3)
O5—Ce1—Cl1[i]	75.12 (14)	O10—P2—Co1	111.9 (2)
O5—Ce1—Cl1	84.36 (13)	O6—P3—Co1	118.5 (2)
O5—Ce1—O1	118.98 (17)	O6—P3—O11	109.6 (3)
O5—Ce1—O2	140.68 (18)	O6—P3—O12	107.2 (3)
O5—Ce1—O3	146.09 (19)	O11—P3—Co1	107.1 (2)
O5—Ce1—O4	75.11 (16)	O12—P3—Co1	111.6 (2)
O6—Ce1—Cl1[i]	80.23 (14)	O12—P3—O11	101.4 (3)
O6—Ce1—Cl1	145.12 (13)	P1—O4—Ce1	135.4 (3)
O6—Ce1—O1	140.00 (18)	P2—O5—Ce1	139.7 (3)
O6—Ce1—O2	71.45 (16)	P3—O6—Ce1	140.8 (3)
O6—Ce1—O3	108.43 (18)	C6—O7—P1	119.4 (4)
O6—Ce1—O4	72.99 (16)	C7—O8—P1	121.1 (5)
O6—Ce1—O5	77.22 (16)	C8—O9—P2	117.3 (5)
P1—Co1—P2	92.63 (7)	C9—O10—P2	121.7 (5)
P1—Co1—P3	89.50 (7)	C10—O11—P3	117.4 (5)
P3—Co1—P2	92.62 (7)	C11—O12—P3	120.6 (5)
Ce1—Cl1—Ce1[ii]	135.83 (6)		

[a] Symmetry codes: (i) y+1/2, x−1/2, z−1/2; (ii) y+1/2, x−1/2, z+1/2.

Section S8. X-ray structure of $[CeCl_2(L_{OMe})_2]$·acetone (**4**)

Table S14. Crystal data an structure refinement details of $[CeCl_2(L_{OMe})_2]$·acetone (**4**).

Crystal data

$C_{22}H_{46}CeCl_2Co_2O_{18}P_6·C_3H_6O$	F(000) = 1168
M_r = 1171.32	D_x = 1.762 Mg m^{-3}
Orthorhombic, $P2_12_12$	Cu $K\alpha$ radiation, λ = 1.54184 Å
a = 20.8164 (2) Å	Cell parameters from 8979 reflections
b = 11.5108 (1) Å	θ = 4.2–76.6°
c = 9.1672 (1) Å	μ = 17.51 mm^{-1}
V = 2196.58 (4) Å^3	T = 150 K
Z = 2	Block, clear red
0.16 × 0.1 × 0.07 mm	

Data collection

XtaLAB Synergy, Dualflex, HyPix	4071 independent reflections
Radiation source: micro-focus sealed X-ray tube, PhotonJet (Cu) X-ray Source	
Mirror monochromator	3945 reflections with $I > 2\sigma(I)$
Detector resolution: 10.0000 pixels mm^{-1}	R_{int} = 0.032
ω scans	θ_{max} = 77.4°, θ_{min} = 4.3°
Absorption correction: gaussian	h = −25-25
CrysAlis PRO 1.171.41.90a (Rigaku Oxford Diffraction, 2020) Numerical absorption correction based on gaussian integration over a multifaceted crystal model Empirical absorption correction using spherical harmonics, implemented in SCALE3 ABSPACK scaling algorithm.	k = −14-13
T_{min} = 0.177, T_{max} = 0.590	l = −11-5
11899 measured reflections	

Refinement

Refinement on F^2	Hydrogen site location: inferred from neighbouring sites
Least-squares matrix: full	H-atom parameters constrained
$R[F^2 > 2\sigma(F^2)]$ = 0.0363<br>$R[F^2$, all data] = 0.0371	$w = 1/[\sigma^2(F_o^2) + (0.0548P)^2 + 3.7797P]$<br>where $P = (F_o^2 + 2F_c^2)/3$
$wR(F^2 > 2\sigma(F^2)$ = 0.0964<br>$wR(F^2$, all data) = 0.0970	$(\Delta/\sigma)_{max}$ = 0.001
S = 1.07	$\Delta\rho_{max}$ = 0.83 e Å^{-3}
4071 reflections	$\Delta\rho_{min}$ = −1.05 e Å^{-3}
265 parameters	Absolute structure: Flack x determined using 1506 quotients [(I+)-(I-)]/[(I+)+(I-)] (Parsons, Flack and Wagner, Acta Cryst. B69 (2013) 249-259).
71 restraints	Absolute structure parameter: −0.011 (3)

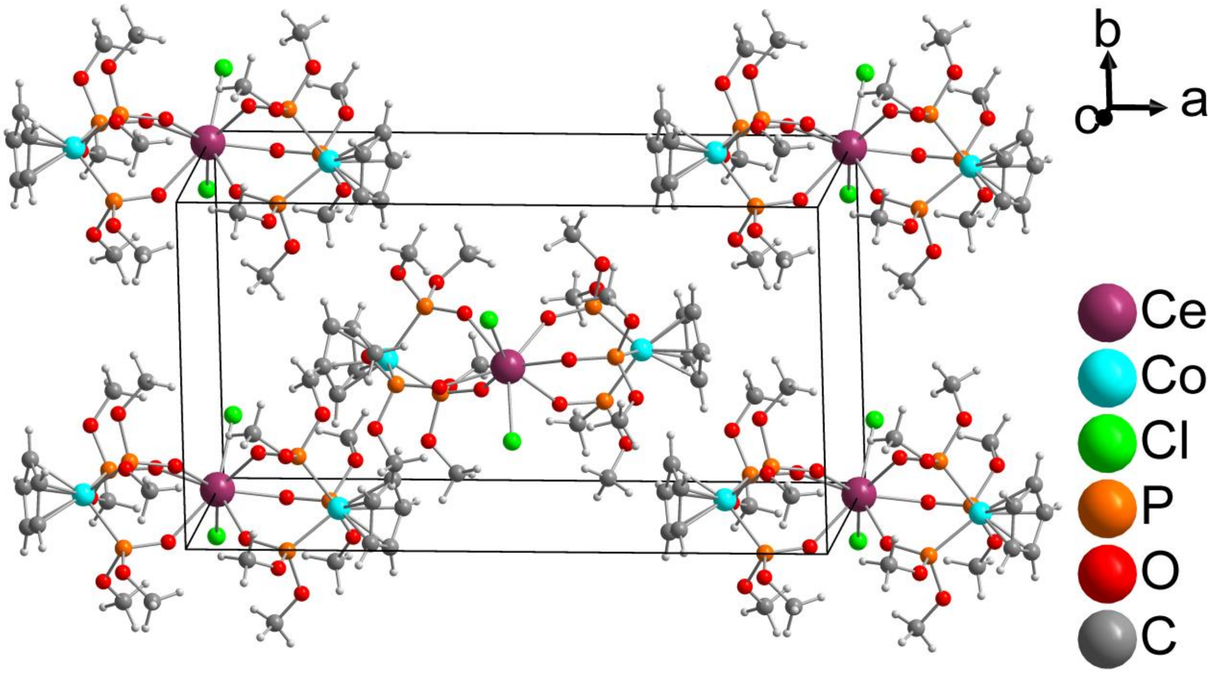

Figure S16. Unit cell of $[CeCl_2(L_{OMe})_2]$·acetone (**4**) with four asymmetric units.

Table S15. Selected bond lengths (Å) and angles (°) of $[CeCl_2(L_{OMe})_2]$·acetone (**4**).[a]

Ce1—Cl1^i	2.7128 (18)	P1—O1	1.516 (5)
Ce1—Cl1	2.7128 (18)	P1—O4	1.566 (8)
Ce1—O1^i	2.329 (5)	P1—O5	1.592 (8)
Ce1—O1	2.329 (5)	P2—O2	1.508 (5)
Ce1—O2	2.336 (5)	P2—O6	1.581 (8)
Ce1—O2^i	2.336 (5)	P2—O7	1.599 (7)
Ce1—O3	2.350 (5)	P3—O3	1.506 (5)
Ce1—O3^i	2.350 (5)	P3—O8	1.572 (7)
Co1—P1	2.149 (2)	P3—O9	1.615 (7)
Co1—P2	2.149 (2)	O4—C6	1.326 (16)
Co1—P3	2.159 (2)	O5—C7	1.387 (14)
Co1—C1	2.075 (9)	O6—C8	1.359 (14)
Co1—C2	2.069 (8)	O7—C9	1.357 (13)
Co1—C3	2.088 (8)	O8—C10	1.399 (11)
Co1—C4	2.083 (9)	O9—C11	1.373 (11)
Co1—C5	2.067 (9)		
Cl1^i—Ce1—Cl1	97.33 (8)	P2—Co1—P1	90.09 (8)
O1—Ce1—Cl1	142.47 (12)	P2—Co1—P3	89.97 (9)
O1—Ce1—Cl1^i	98.46 (14)	O1—P1—Co1	119.6 (2)
O1^i—Ce1—Cl1	98.46 (14)	O1—P1—O4	108.0 (4)
O1^i—Ce1—Cl1^i	142.46 (12)	O1—P1—O5	107.8 (4)
O1^i—Ce1—O1	89.2 (3)	O4—P1—Co1	113.4 (4)
O1^i—Ce1—O2	143.64 (16)	O4—P1—O5	97.7 (7)

O1—Ce1—O2	74.26 (17)	O5—P1—Co1	108.0 (3)
O1—Ce1—O2^{i}	143.64 (16)	O2—P2—Co1	119.3 (2)
O1^{i}—Ce1—O2^{i}	74.26 (17)	O2—P2—O6	108.0 (3)
O1—Ce1—O3	73.08 (17)	O2—P2—O7	108.3 (4)
O1—Ce1—O3^{i}	70.82 (17)	O6—P2—Co1	110.6 (3)
O1^{i}—Ce1—O3	70.82 (17)	O6—P2—O7	102.9 (6)
O1^{i}—Ce1—O3^{i}	73.08 (17)	O7—P2—Co1	106.5 (4)
O2—Ce1—Cl1	78.16 (13)	O3—P3—Co1	119.0 (2)
O2^{i}—Ce1—Cl1	73.12 (12)	O3—P3—O8	108.9 (3)
O2—Ce1—Cl1^{i}	73.12 (12)	O3—P3—O9	108.2 (3)
O2^{i}—Ce1—Cl1^{i}	78.16 (13)	O8—P3—Co1	109.9 (3)
O2—Ce1—O2^{i}	135.9 (2)	O8—P3—O9	101.6 (5)
O2^{i}—Ce1—O3	127.75 (16)	O9—P3—Co1	107.9 (3)
O2—Ce1—O3	73.40 (16)	P1—O1—Ce1	141.0 (3)
O2—Ce1—O3^{i}	127.75 (16)	P2—O2—Ce1	141.8 (3)
O2^{i}—Ce1—O3^{i}	73.40 (16)	P3—O3—Ce1	141.7 (3)
O3—Ce1—Cl1^{i}	146.51 (12)	C6—O4—P1	129.8 (9)
O3^{i}—Ce1—Cl1	146.51 (12)	C7—O5—P1	124.2 (7)
O3^{i}—Ce1—Cl1^{i}	74.98 (12)	C8—O6—P2	125.2 (7)
O3—Ce1—Cl1	74.98 (12)	C9—O7—P2	124.9 (7)
O3—Ce1—O3^{i}	128.4 (2)	C10—O8—P3	125.7 (6)
P1—Co1—P3	91.00 (9)	C11—O9—P3	121.7 (6)

[a] Symmetry code: (i) −*x*+1, −*y*+1, *z*.

Section S9. Coordination polyhedra of cerium in discussed literature complexes.

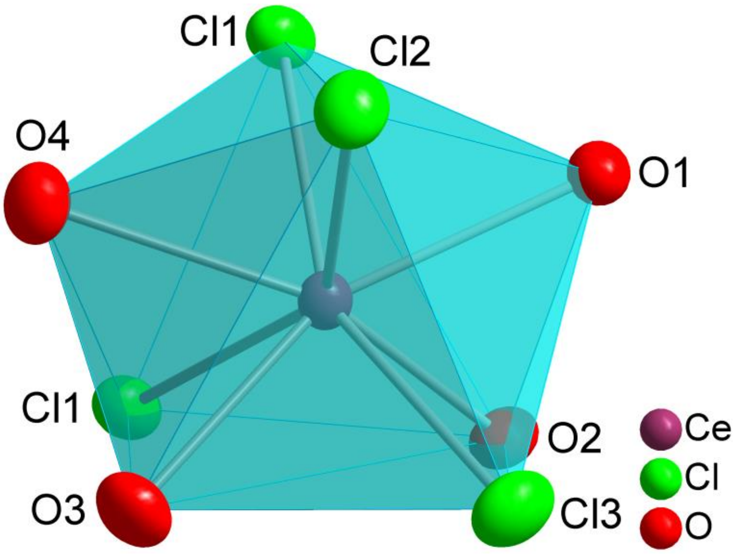

Figure S17. Coordination polyhedron of cerium of $[Ce_2Cl_6(DME)_4]$ [3].

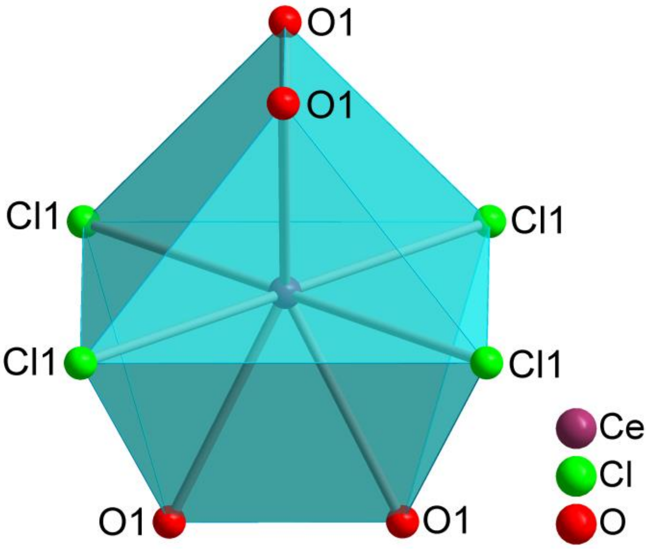

Figure S18. Coordination polyhedron of cerium of $(N(CH_3)_4)_2[CeCl_4(NO_3)_2]$ [4]. The polyhedron here is constructed from attaching a triangular prism to the square face of another one, which in geometry is called a gyrobifastigium.

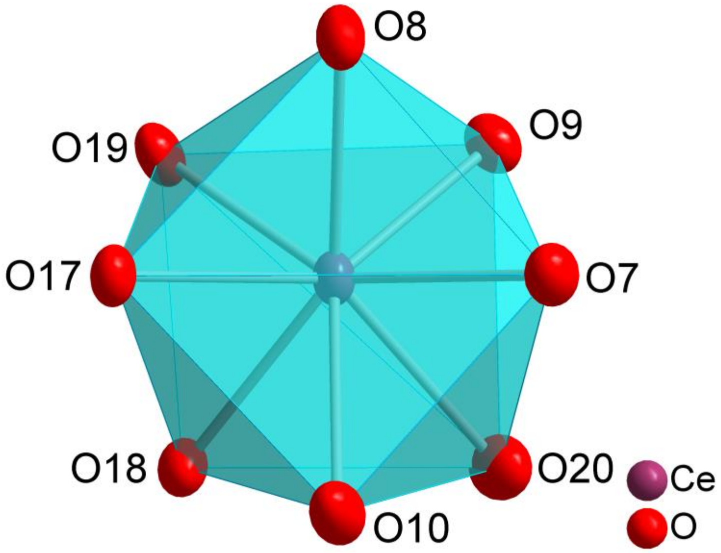

Figure S19. Coordination polyhedron of cerium of $[Ce(L_{OEt})_2(H_2O)_2](OTf)_2{\cdot}H_2O$ [5].

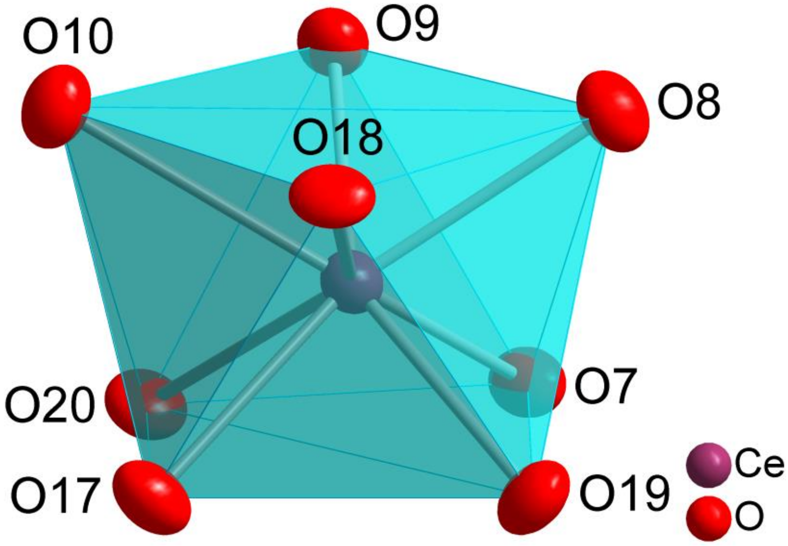

Figure S20. Coordination polyhedron of cerium of $[Ce(L_{OEt})_2(H_2O)_2](BF_4)_2$ [6].

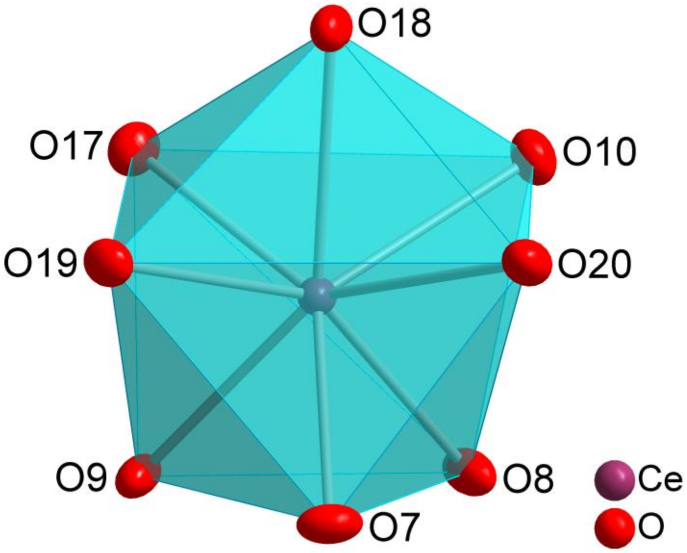

Figure S21. Coordination polyhedron of cerium of [Ce(L_{OEt})$_2$(H_2O)$_2$][L_{OEt}] [5].

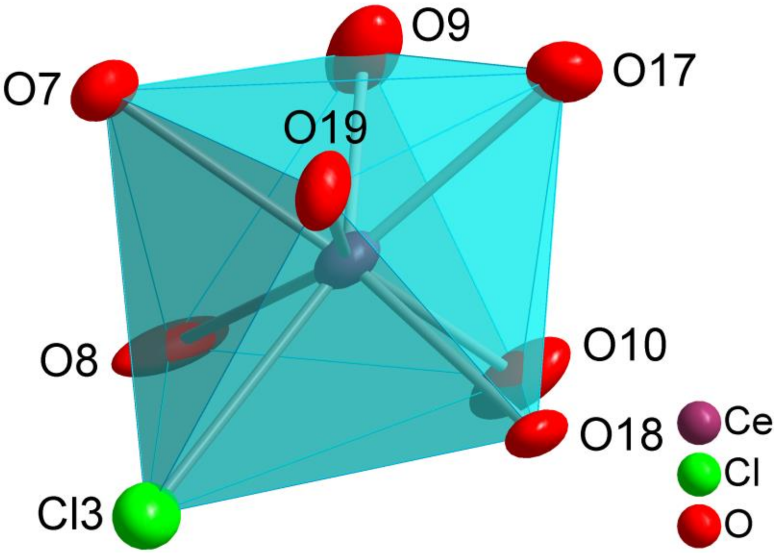

Figure S22. Coordination polyhedron of cerium of [CeCl(L_{OEt})$_2$($OC_6H_3Cl_2$-2,4)] [6].

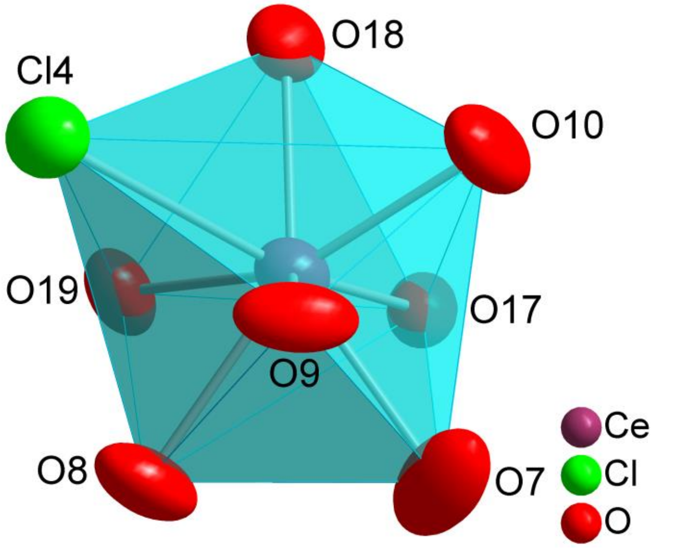

Figure S23. Coordination polyhedron of cerium of [CeCl(L_{OEt})$_2$(O$C_6H_3Cl_3$-2,4,6)] [6].

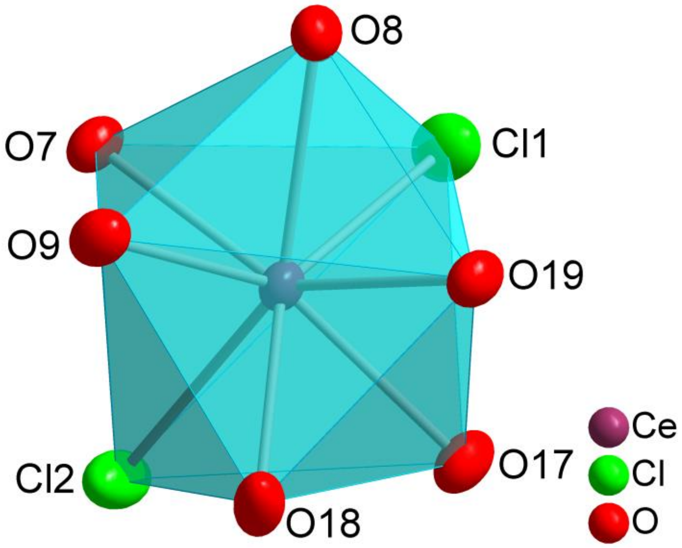

Figure S24. Coordination polyhedron of cerium of [$CeCl_2$(L_{OEt})$_2$] [7].

Section S10. Infrared spectra

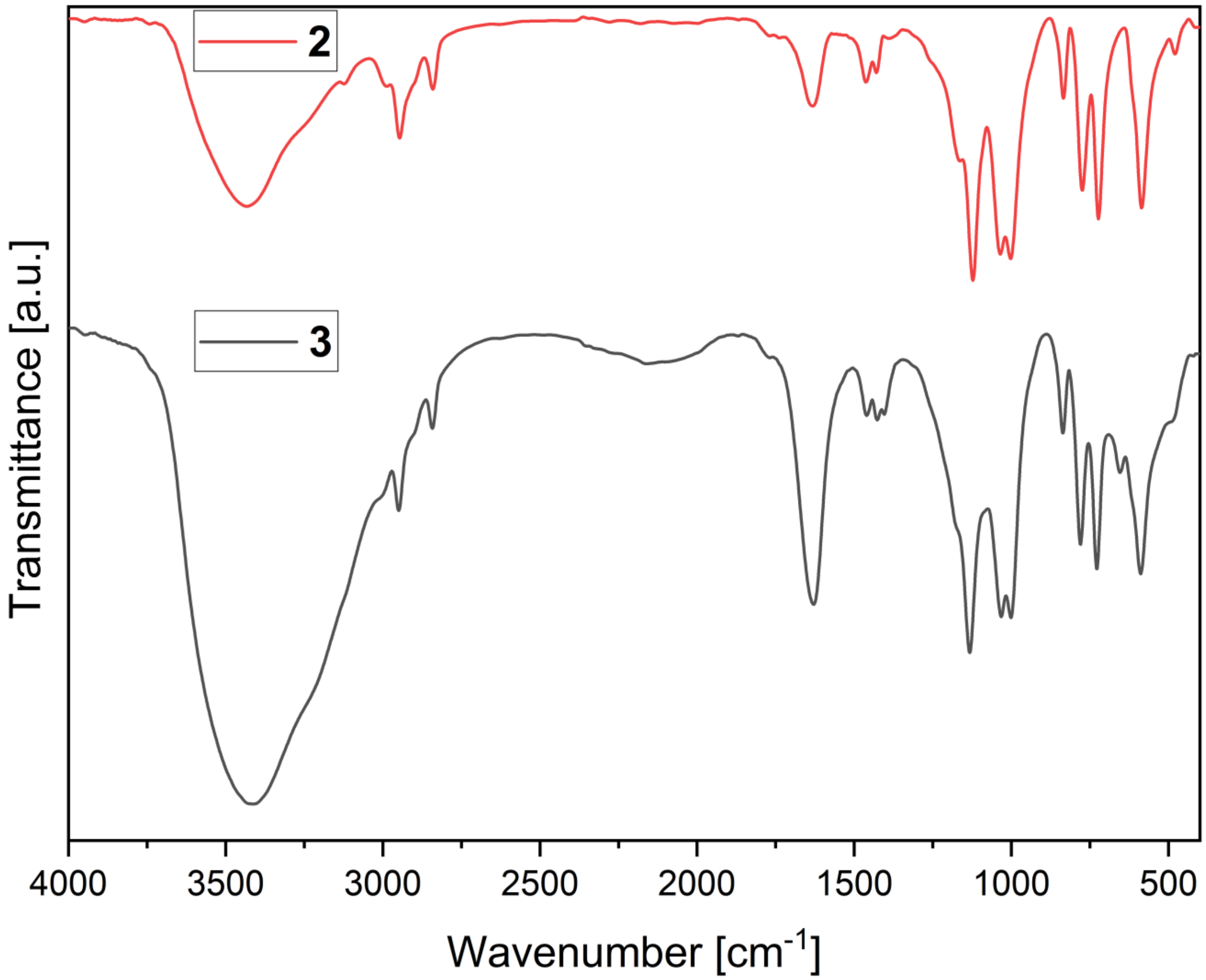

Figure S25. FT-IR spectra of [Ce(L_{OMe})$_2$(H_2O)$_2$]Cl·2.5H_2O (**2**) and [CeCl(L_{OMe})(H_2O)$_3$]Cl (**3**) recorded in the range of 4000–400 cm^{-1} using KBr pellets.

Table S16. Assignment of the FT-IR absorption bands of [Ce(L_{OMe})$_2$(H_2O)$_2$]Cl·2.5H_2O (**2**) including wavenumbers, literature assignments, and relative band intensities. FT-IR band assignments of the Kläui ligand based on literature data [9]. Abbreviations: very weak (vw), weak (w), medium (m), strong (s), very strong (vs), broad (br).

Wavenumber (cm^{-1})	Assignment	Intensity
3434	ν(O–H)	br
2947	ν_{as}(CH_3)	w
2839	ν_s(CH_3)	w
1628	δ(H–O–H)	m
1462	δ_{as}(CH_3)	w
1427	ν(C–C) [Cp]	w
1121	ν_{as}(C–O–(P))	vs
1036	ν_s(C–O–(P))	vs
1003	δ(CH) [Cp]	vs
835	γ(CH) [Cp]	m

775	ϱ(CH_3)	s
723	ϱ(CH_3)	s
586	δ(P═O)	s
480	δ(P–O–C)	vw

Table S17. Assignment of the FT-IR absorption bands of [CeCl(L_{OMe})(H_2O)$_3$]Cl (**3**) including wavenumbers, literature assignments, and relative band intensities. FT-IR band assignments of the Kläui ligand based on literature data [9]. Abbreviations: very weak (vw), weak (w), medium (m), strong (s), very strong (vs), broad (br).

Wavenumber (cm^{-1})	Assignment	Intensity
3416	ν(O–H)	br
2949	ν_{as}(CH_3)	w
2843	ν_s(CH_3)	w
1630	δ(H–O–H)	s
1460	δ_{as}(CH_3)	vw
1427	ν(C–C) [Cp]	vw
1132	ν_{as}(C–O–(P))	s
1034	ν_s(C–O–(P))	s
1001	δ(CH) [Cp]	s
837	γ(CH) [Cp]	w
779	ϱ(CH_3)	s
727	ϱ(CH_3)	s
588	δ(P═O)	s
488	δ(P–O–C)	vw

Section S11. References

1. Kläui, W.; Otto, H.; Eberspach, W.; Buchholz, E. Die Michaelis-Arbusov-Reaktion als Zugang zu neuen Sauerstoff-Tripodliganden des Typs [$C_5R_5M\{P(O)(OCH_3)_2\}_3$]$^-$. Ligandfeldspektroskopische und koordinationschemische Charakterisierung der Liganden. *Chem. Ber.* **1982**, *115*, 1922–1933. https://doi.org/10.1002/cber.19821150522
2. Llunell, M.; Casanova, D.; Cirera, J.; Alemany, P.; Alvarez, S. *SHAPE V2.1. Program for the Stereochemical Analysis of Moelcular Fragments by Means of Continuous Shape Measures and Associated Tools*. University of Barcelona, Spain, **2013.**
3. Baisch, U.; Belli Dell'Amico, D.; Calderazzo, F.; Conti, R.; Labella, L.; Marchetti, F.; Quadrelli, E.A. The mononuclear and dinuclear dimethoxyethane adducts of lanthanide trichlorides [$LnCl_3(DME)_2$]$_n$, n=1 or 2, fundamental starting materials in lanthanide chemistry: preparation and structures. *Inorg. Chim. Acta* **2004**, *357*, 1538–1548. https://doi.org/10.1016/j.ica.2003.11.011
4. Brandt, A.; Kiselev, Yu. M.; Martynenko, L.I. Synthesis and structure of tetramethyl-ammonium tetrachlorodinitratocerate (4). *Zh. Neorg. Khim.* **1981**, *26*, 2950.
5. So, Y.-M.; Li, Y.; Au-Yeung, K.-C.; Wang, G.-C.; Wong, K.-L.; Sung, H.H.Y.; Arnold, P.L.; Williams, I.D.; Lin, Z.; Leung, W.-H. Probing the Reactivity of the Ce═O Multiple Bond in a Cerium(IV) Oxo Complex. *Inorg. Chem.* **2016**, *55*, 10003–10012. https://doi.org/10.1021/acs.inorgchem.6b00480
6. Pham, H.-L.; Rajeshkumar, T.; Wang, L.U.; Ng, Y.H.; Wong, K.-H.; So, Y.-M.; Sung, H.H.-Y.; Lortz, R.; Williams, I.D.; Maron, L.; Leung, W.-H. Synthesis and Reactivity of Redox-Active Cerium(IV) Aryloxide Complexes. *Organometallics* **2023**, *42*, 1094–1105. https://doi.org/10.1021/acs.organomet.2c00569

7. So, Y.-M.; Wang, G.-C.; Li, Y.; Sung, H.H.-Y.; Williams, I.D.; Lin, Z.; Leung, W.-H. A Tetravalent Cerium Complex Containing a Ce=O Bond. *Angew. Chem. Int. Ed.* **2014**, *53*, 1626–1629. https://doi.org/10.1002/anie.201309764
8. Miessler, G.L.;. Fischer, P.J; Tarr, D.A. *Inorganic Chemistry*, 5th ed. Pearson, NJ, USA, **2014**, p. 52.
9. Kläui, W.; Dehnicke, K. Dreikernige Sandwichkomplexe mit Dialkylphosphonat-Brückenliganden: Synthese, NMR-, IR- und Raman-spektroskopische Untersuchungen. *Chem. Ber.* **1978**, 111, 451–468. https://doi.org/10.1002/cber.19781110205
